# Exploring the Impact of 1,8-Diioodoctane on the Photostability of Organic Photovoltaics

**DOI:** 10.1021/acsaem.4c01272

**Published:** 2024-09-13

**Authors:** Rachel C. Kilbride, Emma L. K. Spooner, Elena J. Cassella, Mary E. O’Kane, Khalid Doudin, David G. Lidzey, Richard Jones, Andrew J. Parnell

**Affiliations:** † Department of Chemistry, The University of Sheffield, Dainton Building, Brook Hill, Sheffield S3 7HF, U.K.; ‡ Department of Physics and Astronomy, The University of Sheffield, Hicks Building, Hounsfield Road, Sheffield S3 7RH, U.K.; § The Photon Science Institute, The University of Manchester, Manchester M13 9PY, U.K.; ∥ Department of Materials, The University of Manchester, Sackville Street Building, Manchester M1 3BB, U.K.

**Keywords:** organic photovoltaics, bulk heterojunctions, photostability, 1,8-dioodoctane, solvent additives, crystallinity

## Abstract

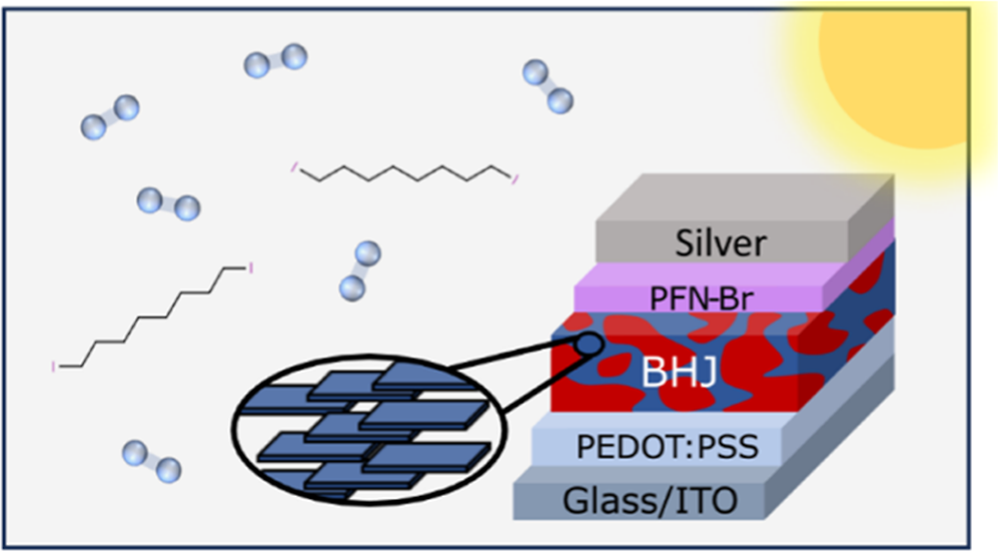

Improving the photostability of the light-harvesting blend film in organic photovoltaics is crucial to achieving long-term operational lifetimes that are required for commercialization. However, understanding the degradation factors which drive instabilities is complex, with many variables such as film morphology, residual solvents, and acceptor or donor design all influencing how light and oxygen interact with the blend film. In this work, we show how blend films comprising a donor polymer (PBDB-T) and small molecule acceptor (PC_71_BM or ITIC) processed with solvent additive (DIO) yield very different film morphologies, device performance, and photostability. We show that DIO is retained approximately 10 times more effectively in ITIC based films compared to PC_71_BM. Unexpectedly, we see that while high volumes of DIO reduce photostability for encapsulated ITIC devices, when oxygen is introduced DIO can improve the lifetime of PBDB-T:ITIC based cells. Here, the addition of 3% DIO doubles the *T*_80_ compared to ITIC based devices without DIO, suggesting that DIO-induced morphological changes interfere with or reduce photo-oxidative reactions.

## 1 Introduction

Over the past decade, the development of nonfullerene acceptor (NFA) materials has increased the power conversion efficiency (PCE) of single-junction organic photovoltaics (OPVs) to over 19%.^1^ Combining this success with the potential for lightweight, flexible, and semitransparent manufacturing establishes OPVs as a promising alternative to conventional solar technologies for a range of applications including building-integrated PV, wearable PV, indoor PV and lightweight, autonomous energy supply.^2−4^ Despite this potential, OPV commercialization has been bottlenecked by a range of issues, including low lifetimes. Unlike with conventional PV, for the niche markets that OPVs are anticipated to occupy stability is considered secondary to functionality, nevertheless it has been estimated a lifetime of around 10 years is still required for grid parity, and stability remains a key area of research.^5^

Many common high efficiency OPV systems still show significant instability, with degradation routes including morphological instability,^6,7^ photo-oxidation^8^ and chemical reactions, such as UV-initiated radical reactions leading to a loss of conjugation.^9^ In some cases, the intrinsic stability of high efficiency NFA based OPVs has been shown to be worse than those using fullerene-based acceptors, with acceptor conformational instabilities leading to severe burn-in under illumination.^10−12^

The incorporation of solvent additives in the active layer casting solution is a common approach used to control the drying dynamics and nanomorphology of solution-processed OPV films.^13^ Additives are selected for a range of reasons, including selective solubility,^14^ desirable surface tension,^15^ or ideal vapor pressure. Solvent additives can be liquid or solid, but the most common are high boiling point small molecules such as 1,8-dioodoctane (DIO), 1,8-octanedithiol (ODT), 1-chloronapthalene (CN) and *N*-methyl-2-pyrrolidone (NMP).^16−18^ DIO in particular has been used extensively with great success, as it is an effective route to control the phase-separation and domain size of both fullerene and NFA-based systems for optimum device performance.^7,19,20^

Despite offering a route to enhance OPV efficiency, the low volatility of DIO and other high boiling point solvent additives makes it difficult to fully remove DIO from the film after deposition. Several studies have reported the development of processing routes to remove significant amounts of DIO from the film such as thermal annealing, high-vacuum exposure, solvent rinsing and light-soaking.^19,21−24^ However, additives are often not removed in their entirety with trace amounts remaining.^21^ Residual DIO in OPV films, even in trace amounts, has frequently been linked to OPV instability, usually via UV-induced reactions of iodooctane radicals and active layer components,^9^ reactions between DIO and charge transport layer materials,^25^ or changes in the vertical distribution of components within the film.^26^ Understanding how to mitigate these instabilities, while maintaining performance improvements, has been hindered by the differing influence of DIO on fullerene and NFA containing OPVs.^9,11^ For example, Song et al.^27^ reported the detrimental impact of high DIO concentration (>1% by volume) on the performance of NFA based cells, that was not replicated in those based on PC_71_BM. The use of DIO as an effective route to control nanomorphology is therefore not universal and often accompanied by a trade-off in long-term operational stability. Improved understanding of the relationships between processing, nanomorphology, performance and photostability and how these differ between fullerene and NFA-based systems is crucial to developing molecularly robust systems that are both highly efficient and photostable.

**Figure 1:**
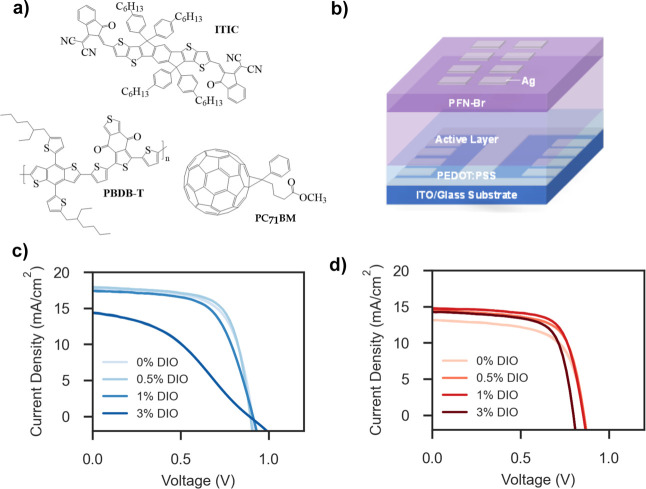
(a) Chemical structures of conjugated donor polymer PBDB-T, fullerene acceptor PC_71_BM and nonfullerene acceptor ITIC. (b) An illustration of the device architecture used in this work. Champion *JV* sweeps of (c) PBDB-T:ITIC and (d) PBDB-T:PC_71_BM devices with varying amounts of DIO additive.

In this work, we explore differences in the photostability of an archetypal polymer:NFA system and an analogous polymer: fullerene system, namely blends of the donor polymer poly[(2,6-(4,8-bis(5-(2-ethylhexyl)thiophen-2-yl)-benzo[1,2-*b*:4,5-*b*′]dithiophene))-*alt*-5,5-(1′,3′-di-2-thienyl-5′,7′-bis(2-ethylhexyl)benzo[1′,2′-*c*:4′,5′-*c*′]dithiophene-4,8-dione)] (PBDB-T) with a surface-functionalized fullerene [6,6]-phenyl-C_71_-butyric acid methyl ester (PC_71_BM) or NFA 3,9-bis(2-methylene-(3-(1,1-dicyanomethylene)-indanone))-5,5,11,11-tetrakis(4-hexylphenyl)-dithieno[2,3-*d*:2′,3′-*d*′]-*s*-indaceno[1,2-*b*:5,6*b*′]dithiophene (ITIC) (Figure 1a) processed with DIO. PC_71_BM and ITIC have been chosen here as examples of a “classic” fullerene and nonfullerene-based acceptor, respectively.

We find that the choice of acceptor in these systems not only influences film morphology, device performance, and stability, but also drastically influences the amount of residual DIO retained in the blend films after processing. Higher concentrations of DIO are found to have different impacts on the resulting thin-film stability for fullerene- or NFA-based systems, in some cases surprisingly improving ambient stability.

## 2 Results and Discussion

### 2.1 Initial Device Performance

The PBDB-T:PC_71_BM and PBDB-T:ITIC systems were first explored in single-junction, OPV devices in a conventional architecture (ITO/PEDOT:PSS/active layer/PFN-Br/Ag, Figure 1b). Full experimental details are included in the Supporting Information. DIO concentration was varied as 0%, 0.5%, 1% or 3% by volume in chlorobenzene. Metrics of fresh devices are summarized in Table 1, with champion current–voltage (*JV*) sweeps for each system depicted in Figure 1c,d and external quantum efficiency measurements shown in Figure S1, and Table S1. Boxplots showing the statistical relationship between the devices can be seen in Figures S2 and S3, with metrics for unencapsulated devices given in Table S2.

**Table 1:** Device Metrics for Encapsulated PBDB-T:ITIC and PBDB-T:PC_71_BM Cells^a^

blend system	DIO concentration [vol %]	*J*_SC_ [mA cm^–2^]	*V*_oc_ [V]	FF [%]	PCE [%]
PBDB-T:ITIC	0	17.5 ± 0.15 (17.8)	0.91 ± 0.00 (0.91)	65.7 ± 0.73 (67.0)	10.3 ± 0.14 (10.6)
	0.5	17.7 ± 0.18 (18.0)	0.89 ± 0.00 (0.90)	69.1 ± 0.33 (69.8)	10.8 ± 0.14 (11.0)
	1	17.1 ± 0.26 (17.5)	0.90 ± 0.01 (0.91)	60.8 ± 2.23 (63.1)	9.23 ± 0.46 (9.94)
	3	14.2 ± 0.87 (14.7)	0.87 ± 0.01 (0.89)	37.6 ± 1.32 (39.7)	4.60 ± 0.32 (5.08)
PBDB-T:PC_71_BM	0	12.7 ± 0.24 (13.2)	0.86 ± 0.02 (0.94)	63.5 ± 2.88 (68.8)	6.86 ± 0.40 (7.33)
	0.5	13.9 ± 0.24 (14.4)	0.85 ± 0.01 (0.86)	69.1 ± 0.50 (70.2)	8.22 ± 0.13 (8.48)
	1	14.2 ± 0.35 (14.8)	0.85 ± 0.00 (0.86)	67.8 ± 2.99 (71.4)	8.12 ± 0.59 (8.84)
	3	13.7 ± 0.32 (14.3)	0.80 ± 0.01 (0.81)	69.5 ± 0.55 (70.2)	7.58 ± 0.22 (7.94)

a An average is given for 10 devices ±1 standard deviation, with the champion value given in brackets.

The results shown here match relative trends and efficiencies seen in other works,^11,27^ in which without solvent additives, the PBDB-T:ITIC cells outperform those based on PC_71_BM, mostly due to the higher short-circuit current density (*J*_SC_) resulting from superior light absorption by the NFA. Upon addition of DIO, device performance is initially increased in both systems. This is likely due to the greater phase purity induced via extended drying times.^19^ As DIO is increased above a volume concentration of 0.5%, PBDB-T:ITIC, PCE values drop due to decreases in *J*_SC_ and FF. The latter of these is in part due to nonstandard, “S-shaped” *JV* curve shapes emerging, as seen in Figure 1c. PBDB-T:PC_71_BM performance remains high even upon addition of 3% DIO, with little statistical difference seen across differing DIO concentrations.

### 2.2 Device Stability

**Figure 2:**
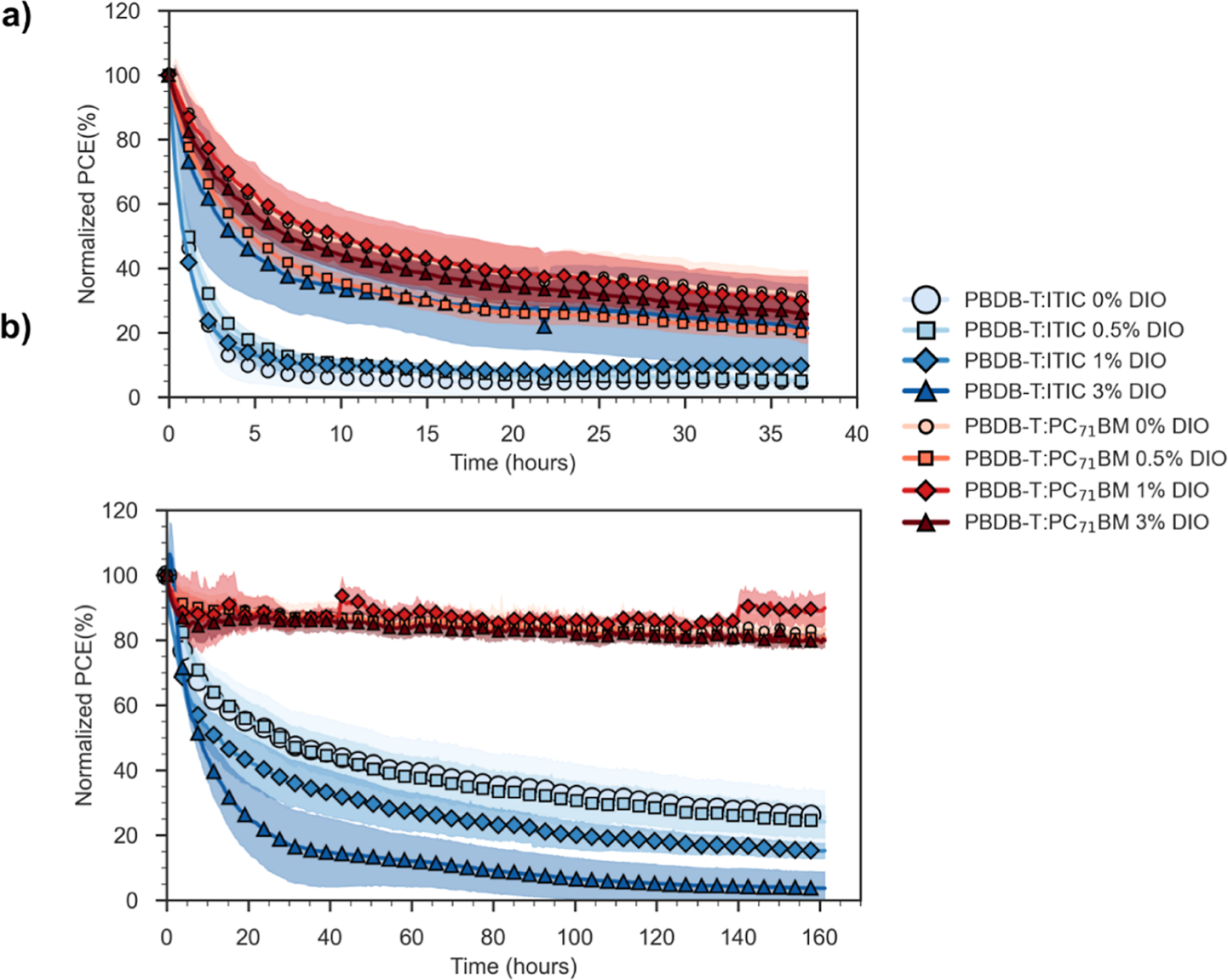
Power conversion efficiency over time for devices illuminated under 1 Sun in ambient conditions, without (a) and with encapsulation (b). In both cases the curves represent the average of four devices across two separate substrates. Shaded area represents ±1 standard deviation.

To examine how DIO influences the photostability of each system, device performance was tracked both under illumination and in the dark. To isolate photostability effects from the influence of oxygen and moisture, we tested both encapsulated cells (where we do not expect O_2_ or H_2_O to affect device stability on the time scale of testing) and unencapsulated cells (which are fully exposed to O_2_ and H_2_O). Tracked device PCE can be seen for cells under 1 sun illumination without (Figure 2a) and with encapsulation (Figure 2b). Data for devices stored in the dark, and further metrics for all devices are shown in Figures S4–S6. Lifetime values, in the form of time taken to reach 80% of the initial PCE (*T*_80_) are summarized in Table S3.

Illuminated, unencapsulated cells (Figure 2a) show rapid burn-in within 10 h of testing, regardless of acceptor or DIO content. PC_71_BM based cells show a slightly reduced rate of burn-in compared to those based on ITIC. This rapid burn-in is driven primarily by decreases in *J*_SC_ (Figure S4b), which is most commonly linked to photo-oxidative breakdown of the absorbing components.^28^ Surprisingly, for both systems higher volumes of DIO have generally yielded improved stability. In PBDB-T:PC_71_BM the differences are relatively small between the 1% and 3% DIO cells. However, this is especially pronounced for the PBDB-T:ITIC system with 3% DIO. The *T*_80_ value for 3% DIO is more than twice that of ITIC-based blends with less DIO.

When the influence of oxygen and moisture are removed by encapsulating devices (Figures 2b and S5), different trends are seen. Here, the PBDB-T:PC_71_BM cells display better stability under illumination than unencapsulated cells, implying the degradation seen in Figure 2a (without encapsulation) occurs due to the effect of both light and oxygen (e.g., photo-oxidation) rather than as a result of light alone. All encapsulated ITIC-based cells exhibit burn-in, but in contrast to unencapsulated cells, the severity of this burn-in is positively correlated with increasing volume of DIO. Regardless of DIO content or encapsulation, the PBDB-T:ITIC based cells are less stable under illumination than those using PBDB-T:PC_71_BM.

Without illumination or the influence of oxygen and moisture (see Figure S6 for encapsulated cells stored in the dark), all PC_71_BM based cells exhibit impressive device stability, varying less than 10% from their original PCE even after over 2000 h of storage. PBDB-T:ITIC cells also demonstrate impressive stability without DIO and with 0.5% DIO. However, at higher DIO concentration (1% and 3%), burn-in is again observed, albeit on slower time scales than under illumination with or without encapsulation.

Therefore, we can see that DIO influences the stability of the OPV systems, with this being dependent on acceptor identity, together with the presence of light, moisture and oxygen, and their combinations. From this device stability data, it is clear that DIO significantly influences the stability of ITIC-based systems. In the absence of O_2_/H_2_O, higher additions of DIO in the active layer solution are correlated with accelerated degradation for ITIC-based systems. Critically, in the presence of O_2_/H_2_O, increased DIO concentration is instead found to reduce the rate of degradation for ITIC-containing cells. Contrastingly, we find that the DIO concentration has little to no influence on the rate of degradation of PCBM-based cells either with or without encapsulation (with or without the presence of O_2_/H_2_O).

### 2.3 UV–Vis Absorption Measurements

**Figure 3:**
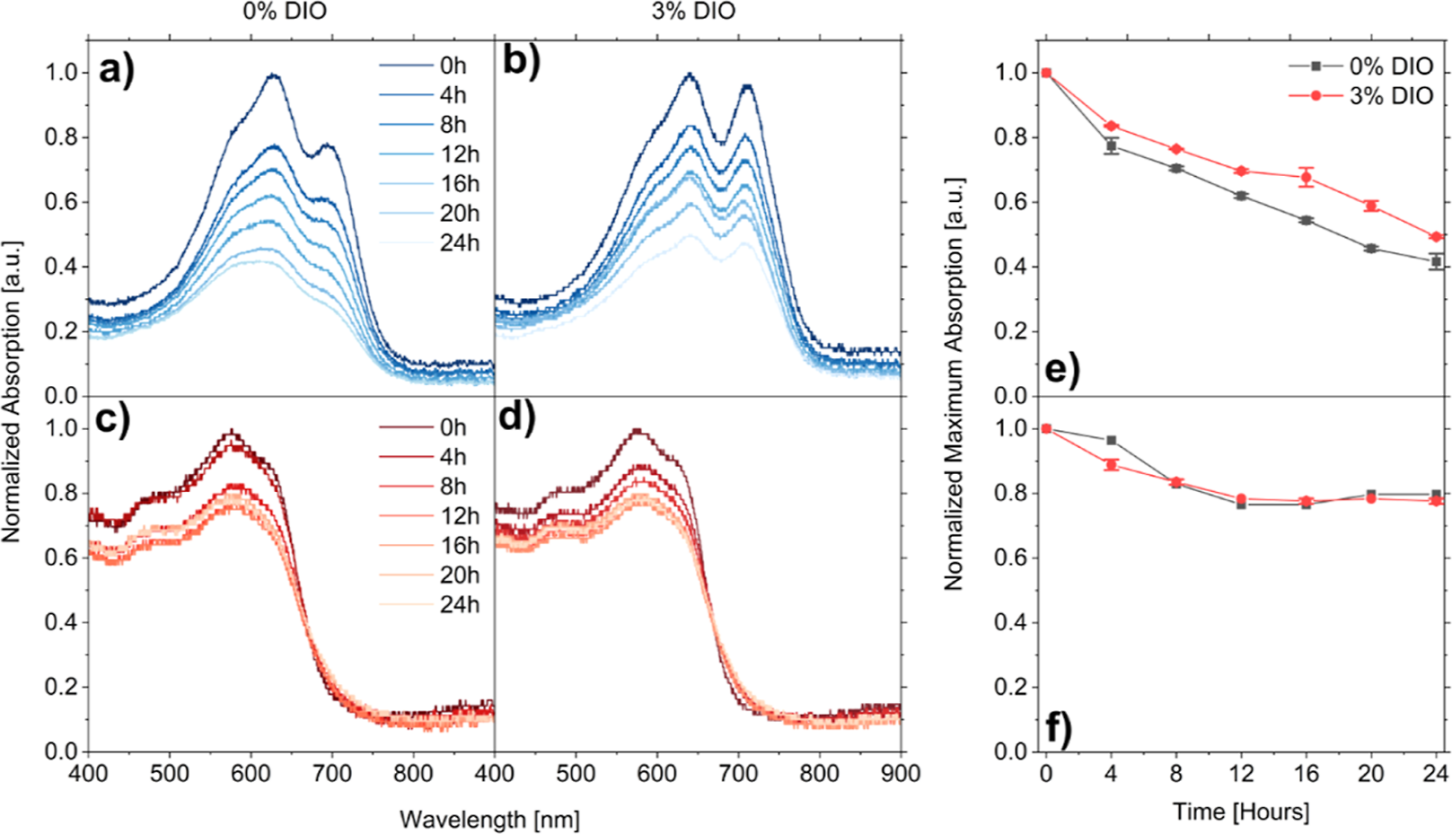
Normalized UV–vis absorption spectra of unencapsulated (a,b) PBDB-T:ITIC and (c,d) PBDB-T:PC_71_BM blend films processed with 0 and 3% DIO, during illumination with 1 Sun simulated solar radiation under ambient conditions. Corresponding normalized maximum absorption as a function of irradiation time for (e) PBDB-T:ITIC and (f) PBDB-T:PC_71_BM blend films.

To further explore the photostability of these systems we aged both neat components and donor:acceptor blend films under 1 Sun simulation solar irradiance, as for device stability tests. Figure S11 shows photographs of neat component films after illuminated aging for up to 96 h. Photo-oxidation reactions of active layer components usually lead to loss in conjugation and corresponding loss of photoabsorbing ability (commonly referred to as “photobleaching”). DIO concentration appears to have a significant effect on the rate of photobleaching, particularly for neat PBDB-T and ITIC films and PBDB-T:ITIC blends. Here, films appear visibly less photobleached when processed with higher concentrations of DIO. To understand the extremes of this effect we performed UV–vis absorption spectroscopy measurements of unencapsulated blend films processed with 0% and 3% DIO during 1 Sun simulated solar irradiation under ambient conditions for 24 h (Figure 3a–d). To quantitatively compare photodegradation rates, we tracked the normalized maximum absorption for each system (Figure 3e,f). Additionally, we include normalized UV–vis absorption and photographs of neat components films after aging (Figures S7–10).

From Figures S7–S11 it is clear that some neat components have better photo-oxidative stability than others. ITIC undergoes rapid photo-oxidative bleaching, with loss of absorption for all concentrations of DIO (Figures S8 and S11a). The lowest amount of photobleaching is observed at the highest concentration of DIO, reflecting the unencapsulated device stability measurements (Figure 2a). Despite this, all pure ITIC films bleached completely within 24 h of illumination, regardless of DIO content. Small amounts of photobleaching are seen for pure PBDB-T (Figures S10 and S11b), again appearing slightly slowed by higher concentrations of DIO. In contrast, pure PC_71_BM films (Figures S9 and S11c) showed very little photobleaching.

The stability of the neat components is reflected in the blend absorption, whereby at all DIO concentrations bleaching is greater for PBDB-T:ITIC films (Figure 3e) compared to PBDB-T:PC_71_BM (Figure 3f). Critically, processing with 3 vol % DIO is found to suppress absorption-loss for PBDB-T:ITIC blend films. In contrast, DIO concentration does not appear to significantly influence the photo stability of neat PC_71_BM films (Figures S9 and S11c) or PBDB-T:PC_71_BM blend films (Figures 3f and S11e). These results mirror those of the unencapsulated, illuminated aging experiments, devices and neat components (Figures 2a and S8–S11).

### 2.4 Tracking the Removal of DIO

**Figure 4:**
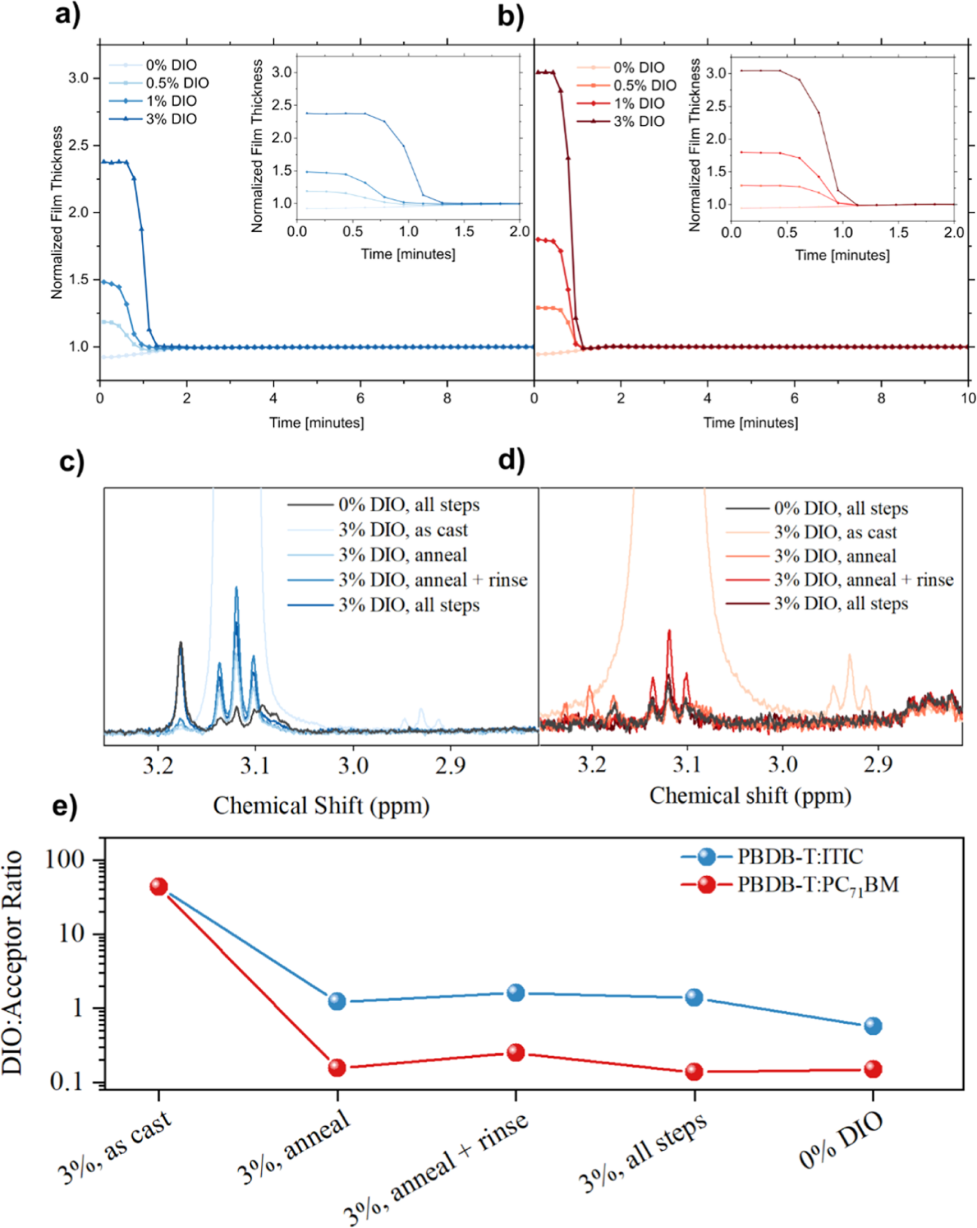
Dynamic spectroscopic ellipsometry during isothermal annealing at 160 °C of (a) PBDB-T:ITIC and (b) PBDB-T:PC_71_BM blend films processed with 0–3 vol % DIO. ^1^H NMR spectra of (c) PBDB-T:ITIC and (d) PBDB-T:PC_71_BM blend films after exposure to various processing steps. (e) Calculated molar ratios of DIO/acceptor for each blend film after processing.

To further understand the role of DIO influences on stability, we have characterized the amounts of DIO left in each active layer film after processing. The presence of residual DIO in the OPV film after processing can result in detrimental UV-initiated radical reactions which leads to a loss in aromatic conjugation and resulting loss in photoabsorption over time.^21^ Due to the high boiling point of DIO, high temperature annealing is necessary to remove the additive from the as-cast thin-films. To investigate the effectiveness of the fabrication protocols used in this work at removing DIO from PBDB-T:ITIC and PBDB-T:PC_71_BM blend films, we performed spectroscopic ellipsometry measurements to track the removal of DIO during isothermal annealing using the experimental setup illustrated in Figure S12. To replicate the active layer annealing step during device fabrication, films were isothermally annealed at 160 °C for 10 min in a nitrogen-filled chamber. Figure 4a,b show the variation in the relative thickness of PBDB-T:ITIC and PBDB-T:PC_71_BM blend films processed from chlorobenzene with different concentrations of DIO normalized to the final film thickness after annealing. We find that processing films with larger concentrations of DIO results in initially thicker as-cast films for both systems due to the greater solvent content. During the initial annealing period, the thickness of films processed with DIO decreases; a process which we attribute to the removal of DIO from the film via evaporation. Contrastingly, films processed without DIO undergo a small increase in film thickness, attributed to thermal expansion of the film during annealing.

Although ellipsometry measurements demonstrate that thermal annealing is an effective route to remove DIO, it is difficult to quantify the amount of DIO removed by annealing treatments and if it is removed in its entirety. Moreover, further device processing steps, such as vacuum exposure during thermal evaporation of the device contacts, may influence the residual DIO content. To investigate this, we performed ^1^H NMR spectroscopy measurements of blend films after each processing step involved in device fabrication. Here “as cast” films have had no processing after spin-coating, “annealed” films were annealed at 160 °C for 10 min (as with the active layers in devices), “rinsed” films were spin rinsed with pure methanol (to imitate the deposition of the PFN-Br in methanol), and “all steps” films were exposed to an anneal, rinse and high vacuum exposure (the latter of which mimics the vacuum exposure that occurs during thermal evaporation of the top Ag device contact). These films were then dissolved in chlorobenzene which was left to evaporate before the residual solid was extracted and dissolved in *d*-chloroform for ^1^H NMR spectroscopy.

Full NMR spectra are shown in Figures S13–S16, with the DIO peaks (∼3.20 ppm^21^) in each blend shown in Figure 4c,d. It is clear here that DIO is present in all samples, under all processing conditions, albeit at much lower intensities when processing is applied. Notably, DIO is even detectable in the films fabricated with a 0% DIO solution, likely due to difficulties in completely removing the solvent vapor from the atmosphere inside the glovebox and spin-coater. This is an interesting observation and highlights how the atmosphere in what is thought to be a highly controlled environment can become contaminated with other materials used in such a chamber, which may in turn alter device performance.

While the absolute DIO concentration cannot be determined, the ratio of DIO/acceptor peaks can be used to compare relative DIO concentrations between samples.^21^ Here the peak area of the relevant components, shown in Figure S17, can be used to calculate relative molar concentration. Further details of this calculation are provided in Supporting Information Note 1, with the molar concentrations depicted in Figure 4e. Here, the residual DIO in 3% DIO films is more than a magnitude higher after all processing steps in PBDB-T:ITIC films compared to PBDB-T:PC_71_BM, suggesting the NFA based system retains DIO to a greater extent. This apparent difference in ease of DIO removal has not been reported explicitly elsewhere, and may explain a number of differences in performance, stability and morphology between the two systems. Other works have seen differing changes in stability upon annealing for fullerene- and NFA-based DIO containing films, potentially arising from differences in the retained residual DIO.^9^ Given the similar solubilities of PC_71_BM and ITIC in DIO,^27^ we note that this effect is likely driven by other factors such as differing acceptor molecular structure and packing motifs,^29^ solid content differences or differences in solution viscosity.^13^

### 2.5 Film Crystallinity

**Figure 5:**
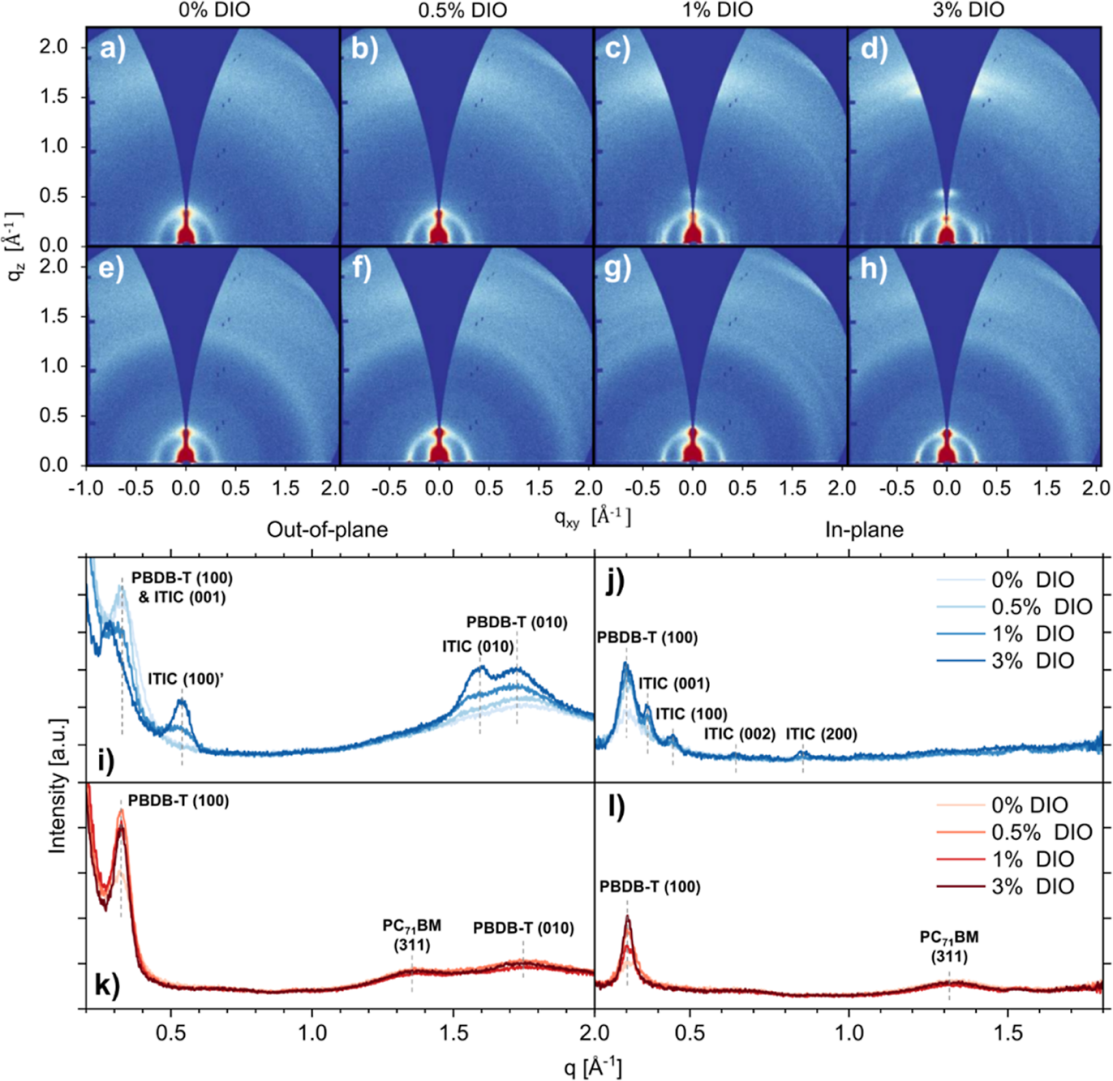
2D GIWAXS patterns of fresh (a–d) PBDB-T:ITIC and (e–h) PBDB-T:PC_71_BM blend films processed with 0, 0.5, 1, and 3 vol % DIO. Corresponding out-of-plane and in-plane 1D azimuthally integrated intensity profiles of (i,j) PBDB-T:ITIC and (k,l) PBDB-T:PC_71_BM blend films.

To probe the impact of DIO on molecular packing and the crystallinity of the blend films, we performed grazing incidence wide-angle X-ray scattering (GIWAXS) and atomic force microscopy (AFM). In Figure 5, we show 2D GIWAXS patterns and corresponding q-dependent intensity profiles of fresh PBDB-T:ITIC and PBDB-T:PC_71_BM blend films processed with each concentration of DIO. GIWAXS data of neat reference films of ITIC, PC_71_BM and PBDB-T which were spin-coated with the same DIO additive concentrations as blend films are included in the Supporting Information (Figures S18–S20), along with angle-dependent intensity profiles (also referred to as pole figures, Figure S21).

The 2D scattering of PBDB-T:ITIC blend films processed without DIO consists of two distinct scattering features at *q* = 0.32 Å^–1^ (*d* = 2π/*q* = 19.5 Å) and *q* = 1.75 Å^–1^ (*d* = 3.58 Å). The length scale at low *q* has been previously indexed as a superposition of ITIC (001) backbone stacking and PBDB-T (100) lamellar stacking with the higher *q* feature attributed to PBDB-T (010) π–π stacking, as labeled in Figure 5i.^26,30^ With increasing DIO concentration, several additional peaks appear in the in-plane direction at *q* = 0.37, 0.45, 0.64, and 0.86 Å^–1^ (*d* = 17.0, 14.1, 9.79, and 7.31 Å), consistent with the (001), (100), (002) and (200) reflections observed for the neat ITIC film (Figure S18). Additional peaks are also observed in the out-of-plane direction at *q* = 0.54 Å^–1^ (*d* = 11.7 Å) and *q* = 1.58 Å^–1^ (*d* = 3.98 Å) relating to the (001)’ edge-on lamellar stacking and π–π stacking (010) of ITIC respectively. Such ITIC packing has been observed elsewhere and indicates a bimodal lamellar arrangement.^30^ The appearance of ITIC crystalline peaks and increase in scattering intensity with higher DIO concentrations shows that DIO processing results in enhanced ITIC crystallinity. We also observe an increase in in-plane intensity and a decrease in out-of-plane intensity of the PBDB-T lamellar stacking packing, suggesting a more pronounced face-on molecular orientation of the polymer with DIO processing. This is seen more clearly as an increase in the angle-dependent intensity profiles in the in-plane direction for neat PBDB-T films processed with increasing DIO content (Figure S21c) and is replicated in the PBDB-T:ITIC blend films, albeit to a lesser extent (Figure S21d).

This observed change in PBDB-T molecular orientation is in agreement with results reported elsewhere,^26^ and is likely related to the increase in initial device performance of PBDB-T:ITIC blends observed at low DIO concentrations (0.5%), due to enhanced charge transport perpendicular to the electrodes. AFM measurements of PBDB-T:ITIC films show an increase in film roughness with increasing DIO content (Figure S26a, Table S5), and the development of a large-scale nanomorphology in agreement with our previous morphological studies.^7^ The drop in device performance at higher DIO concentrations for PBDB-T:ITIC is therefore likely due to excessive ITIC crystallization and phase separation with PBDB-T.

For PBDB-T:PC_71_BM blends films processed without DIO, the same PBDB-T lamellar stacking and π–π stacking peaks are observed as for PBDB-T:ITIC blend films, in addition to a broad scattering feature at *q* = 1.33 Å^–1^ (*d* = 4.71 Å), which has previously been attributed to the (311) reflection of the hexagonal close-packed C_70_ lattice.^31,32^ The isotropic and diffuse nature of this feature indicates that PC_71_BM is weakly ordered in the film. Processing with DIO appears to have a minimal effect on PC_71_BM ordering, with only the PBDB-T lamellar peak increasing in scattering intensity, indicating enhanced molecular order of the polymer (as seen for PBDB-T:ITIC films). This is in agreement with the small increase in device performance observed for PBDB-T:PC_71_BM based devices processed with DIO. AFM measurements of PBDB-T:PC_71_BM blend films processed with DIO show only subtle changes in film nanomorphology with comparable RMS roughness values of ∼1 to 2 nm (Figure S26b, Table S5).

The different degrees of order observed in films based on ITIC and PC_71_BM may be linked to the differing levels of residual DIO seen in the ^1^H NMR spectroscopy measurements. ITIC and other NFAs are known to form various aggregates via π–π stacking,^33,34^ yielding long-range, closely packed crystallites, typically with high levels of face-on orientation. This can be seen in Figure 5, with the effect enhanced by processing with higher concentrations of DIO. We speculate that either the closely packed crystallites are associated with increased trapping of DIO in the ITIC-based films, or that they are a direct result of such trapping yielding slower drying times. In contrast, while PC_71_BM also forms aggregates, it yields far less orientated or long-range structures, which are not significantly influenced by DIO content. Such differences illustrate why excess DIO has such a different effect on initial device performance between the fullerene- or NFA-based systems. As 3% DIO generates excessive crystallization in ITIC films but has little to no effect on PC_71_BM ordering.

To understand how blend film crystallinity changes over time, and how it is influenced by DIO content, we performed ex situ GIWAXS measurements of PBDB-T:ITIC and PBDB-T:PC_71_BM blend films after prolonged aging under 1 Sun simulated solar irradiation at 8 h intervals for a period of 24 h (Figures S22 and S24). Control ex situ GIWAXS measurements were also performed after storage in the dark for 2 weeks under ambient conditions (Figures S23 and S25). For PBDB-T:ITIC blend films, there is generally a decrease in scattering intensity of both PBDB-T and ITIC scattering peaks due to a loss of conjugation as observed in UV–vis measurements (Figure S22). Notably, the (001)’ scattering feature *q* = 0.54 Å^–1^ that corresponds to edge-on lamellar stacking of ITIC increases in intensity during irradiation under ambient conditions. We suspect this results from a rearrangement of ITIC molecules during relaxation to a thermodynamic equilibrium, driven by the greater mobility of active layer components mediated by the residual DIO and the elevated temperature during solar irradiation.

For PBDB-T:PC_71_BM blends (Figures S24 and S25), we observe a similar loss in scattering intensity of the PBDB-T (100) lamellar peak during illumination that is suppressed during storage in the dark. The increase in intensity of the PC_71_BM scattering feature at *q* = 1.33 Å^–1^ during illumination suggests the fullerene molecules undergo some aggregation under light stress. In general, PC_71_BM based blends appear less prone to photodegradation when compared to analogous ITIC blends. This is most likely due to the greater intrinsic photostability of PC_71_BM and the lower amount of residual DIO retained after film processing; a finding in agreement with the device stability results discussed above.

## 3 Experimental Methods

### 3.1 Materials

All solvents were purchased from Sigma-Aldrich. ITIC, PBDB-T (M1003, *M*_w_: 90,311 g/mol) and PC_71_BM were purchased from Ossila. Solid materials were stored in the glovebox but weighed out in air, with solvents added inside a nitrogen-filled glovebox.

### 3.2 Organic Solar Cell Fabrication

Devices were manufactured on 8-pixel, prepatterned ITO substrates (Ossila, batch S211). Substrates were cleaned by stepwise sonication in dilute Hellmanex III (Ossila), deionized water and isopropyl alcohol, with each step lasting 10 min in a water bath held at ∼50 °C. Following cleaning the substrates were dried with a N_2_ gun and exposed to UV–ozone for 15 min. PEDOT:PSS (Ossila, Al 4083) was filtered through a 0.45 μm PVDF microdisc filter before use. A ∼30 nm PEDOT:PSS layer was coated via dynamic spin coating in air at 6000 rpm for 30 s. The ITO was exposed via patterning using a cotton bud dipped in water. The films were then annealed at 110 °C for 15 min in air and transferred to a N_2_ filled glovebox, followed by a further anneal at 110 °C for 15 min. Active layer solutions were made at either 15 mg/mL (PBDB-T:PC_71_BM) or 18 mg/mL (PBDB-T:ITIC), at a weight ratio of 1:1 in chlorobenzene and stirred overnight at 60 °C before use. The relevant amount of DIO for each solution was added at the same time as the chlorobenzene, usually from a stock solution. PBDB-T:PC_71_BM films were spin coated dynamically at 1000 rpm for 40 s, PBDB-T:ITIC films were spin coated dynamically at 2000 rpm for 40 s, both to achieve a film thickness of ∼100 nm. All films were annealed at 160 °C for 10 min. PFN-Br (Ossila) solutions were made at 0.5 mg/mL in methanol and stirred overnight without heating before use. PFN-Br films were spin coated dynamically at 3000 rpm for 30 s without anneal. The ITO was exposed by scraping the films off using a razor blade. An Ag cathode (100 nm) was then thermally evaporated at a pressure of 2 × 10^–6^ mbar through a shadow mask with a defined pixel area of 4 mm^2^. Following electrode deposition, some devices were encapsulated using a UV curable epoxy (Ossila), which was dropped onto the substrate, topped with a glass slide, and cured for 15 min under a lamp at ∼365 nm. All layer thicknesses were measured using a Bruker DetakXT profilometer. *JV* sweeps were measured using a Newport 92251A-1000 solar simulator which had been calibrated using a certified silicon reference cell. Devices were illuminated through an aperture mask with each pixel area restricted to 0.0256 cm^2^. External quantum efficiency measurements were taken using a Newport QuantX-300 Quantum Efficiency Measurement System, using a 100 W xenon arc lamp and Oriel CS130B monochromator. Integrated *J*_SC_ values were obtained using an interpolated AM1.5G spectrum available from NREL.

For characterization measurements, blend films were prepared following the same device film protocols outlined above. Neat ITIC, PC_71_BM and PBDB-T films were prepared at a solid concentration of 15 mg/mL in chlorobenzene using the same DIO concentrations and annealing procedures as for blend films.

### 3.3 Organic Solar Cell Stability Testing

Devices aged under dark ambient conditions were kept in the dark and tested periodically during storage in a laboratory maintained at 17–22 °C and 55–60% relative humidity. Devices illuminated under ambient conditions were kept inside an Atlas Suntest CPS+ tester, maintained at a temperature of 41–44 °C and a relative humidity of 23–27%. These devices were kept at open circuit voltage and tested approximately every 20 min without an aperture mask. Devices were mounted inside the Atlas using an Ossila test board and as such were illuminated through the ITO.

### 3.4 UV–Vis Absorption Spectroscopy

Samples for UV–vis absorption were prepared on PEDOT:PSS coated ITO/glass substrates. UV–vis absorption spectra were determined from the change in transmission of light directed through the sample from a deuterium/tungsten-halogen lamp. Transmitted light was detected using a fiber-coupled Ocean Optics CCD-detector. Data was acquired using OceanView software. Each data set is an average of two samples. Absorption spectra taken during aging measurements were normalized to the maximum absorption value of the initial spectra.

### 3.5 Spectroscopic Ellipsometry

Spectroscopic ellipsometry (M2000v, J.A. Woollam Co.) was used to measure the drying dynamics of the blend films, over time as they were annealed at 160 °C using a Linkam heating/cooling stage (THMS600). Samples were prepared on silicon substrates with a native surface oxide layer. During isothermal annealing, samples were enclosed in a chamber with continuous nitrogen flow and transparent windows to allow transmission of the polarized incident and reflected ellipsometry beams (a diagram of the in situ ellipsometry setup is included in the Supporting Information, Figure S12). Using CompleteEASE software, a Cauchy model was fitted to Ψ (the ratio of the incident and reflected amplitudes) and Δ (the ratio of the phase difference of the incident and reflected light) over the wavelength range where the films are optically transparent (850–1000 nm). The blend films were rapidly heated from 25 to 160 °C at a rate of 90 °C/min and left for 10 min with Ψ and Δ recorded as a function of annealing time to track the evolution of film thickness.

### 3.6 ^1^H NMR Spectroscopy

NMR spectroscopy samples were prepared by fabricating films on ITO glass with the same film thicknesses used in devices and following the same device fabrication protocols. “As cast” samples had no further processing and “annealed” samples were annealed for 160 °C for 10 min. “Annealed + rinse” samples were rinsed via dynamic deposition of methanol at 3000 rpm following the anneal. “All steps” samples were placed inside the evaporator (following an anneal and rinse) and subjected to the same pump down and vacuum time as devices during an Ag evaporation. After processing, approximately ∼10 films per condition were dissolved in chloroform with the solution then left to evaporate over ∼24 h. Following this, the solid (∼5 mg) was redissolved in 600 μL of *d*-chloroform, with this then characterized via ^1^H NMR spectroscopy.

### 3.7 GIWAXS

GIWAXS measurements were performed using a Xeuss 2.0 SAXS/WAXS X-ray scattering instrument (Xenocs) equipped with a liquid gallium MetalJet X-ray source (Excillum). In this experiment, a collimated X-ray beam is incident on the sample surface at a grazing angle of 0.16° and scattered X-rays were collected using a vertically offset Pilatus 1 M area detector (Dectris) positioned ∼300 mm from the sample center. The sample to detector distance was calibrated using a silver behenate standard measured in transmission geometry. The entire flight path including the collimation tubes and sample chamber were held under vacuum to minimize background air scatter. Detector images were corrected, reshaped and reduced using python code which relies on pyFAI and pygix libraries.^35^ Azimuthally integrated *q*-dependent intensity profiles were performed across various azimuthal angle (χ) ranges; out-of-plane (−20° < χ < 20°) and in-plane (60° < χ < 90°). Χ-Dependent intensity profiles were performed across the full azimuthal angle range between *q* = 0.2 Å^–1^ and *q* = 0.4 Å^–1^.

### 3.8 AFM

AFM measurements were performed on blend films using a Dimension 3100 (Veeco) microscope, equipped with a Nanoscope 3A feedback controller. Scout 350 RAl (NuNano) cantilevers were used with a resonant frequency of 350 kHz and spring constant of 42 N m^–1^. Images were processed using Gwyddion software (version 2.60).^36^ RMS roughness were extracted using the statistical quantities tool in Gwyddion.

## 4 Conclusion

In this work, we have compared OPVs based on ITIC and PC_71_BM in blends with the donor polymer, PBDB-T. We find that processing with the solvent additive DIO yields higher crystallinity for ITIC-based devices, but poorer initial device performance when used in excess. In contrast, DIO does not significantly influence the crystallinity of PC_71_BM in blend films. While ellipsometry measurements show that thermal annealing of the active layer during device fabrication is effective at removing this DIO from films, ^1^H NMR spectroscopy clearly shows that significant amounts of DIO remain in PBDB-T:ITIC based films. Notably, we find that an order of magnitude more DIO is retained in ITIC-based thin-films than for PC_71_BM-based films. This is a novel finding that may explain the differences in DIO impact between PCBM and ITIC, seen in other works.^9,27^

When these devices are aged, we see a complex relationship between DIO content and stability. In the dark, without the influence of oxygen or moisture, PBDB-T:PC_71_BM based devices are stable, regardless of DIO content. In contrast, PBDB-T:ITIC devices are only stable in the dark with low concentrations of DIO, meaning that ITIC is either more susceptible to DIO induced degradation, or that higher residual DIO concentrations in these films plays a role in the dark stability; the latter effect being likely due to higher solvent contents yielding higher molecular mobility.^19^

Under illumination (without the presence of oxygen or moisture) we see significant degradation in devices based on PBDB-T:ITIC, but not in those based on PBDB-T:PC_71_BM. Other studies have seen these differences,^9,11^ and the poor intrinsic stability of ITIC^37^ compared to PC_71_BM, but in this work the inter-related degradation factors are clearer when DIO retainment, and differing stress conditions are examined. For example, it is possible that the higher residual DIO concentration in ITIC based films may exacerbate their intrinsic instability, especially considering evidence elsewhere that the impact of DIO can differ depending on chemical structure of the components.^38^

When oxygen and moisture are introduced via the removal of encapsulation, both PC_71_BM and ITIC based devices demonstrate rapid burn-in. However, we find that ITIC based devices processed with higher concentrations of DIO demonstrate a surprising improvement in photostability, a finding not seen elsewhere.

The exact origin of this improvement in photostability is difficult to establish and may be due to a myriad of factors including the acceptor identity, initial DIO addition, and residual DIO content. Aspects such as crystallinity, size of domains, vertical stratification or DIO induced reactions may all contribute to the differences seen here between PBDB-T:ITIC and PBDB-T:PC_71_BM stability. It is likely that there is a stabilizing mechanism competing with illumination induced (and DIO accelerated) reactions. A potential explanation could be related to increases in crystallinity in ITIC based films when processing with higher concentrations of DIO. We suspect that such densely packed structures may lead to decreased rates of oxygen and moisture ingress, thereby reducing the rate of degradation as observed elsewhere.^28,39−41^

We suggest therefore that solvent additives are used carefully when optimizing OPVs. If solvent additives are used during processing, we propose that the use of benign, low boiling point solvents that can be removed fully from the film during standard device fabrication protocols will help avoid detrimental chemical reactions with device components. A promising alternative is the use of solid additives, which have grown in prominence in recent years.^42^ It has been shown that volatile solid additives can be completely removed from active layer films,^43^ preventing instabilities due to residual amounts, and involatile variants are generally designed to remain in the films beneficially, usually imparting improved lifetimes.^44−46^

Furthermore, strategies to improve the intrinsic photostability of NFAs while aiming for stable morphologies are required. Such morphologies may be close packed to reduce the ingress of moisture and oxygen, or simply kinetically quenched to reduce phase separation, and in both cases should be balanced to create OPVs that are simultaneously efficient and long-lasting.

## Supporting Information Available

Additional data and information concerning organic solar cell devices, UV–vis absorption spectroscopy, spectroscopic ellipsometry, ^1^H NMR spectroscopy, GIWAXS and AFM (PDF)
