# Supplementary material for: Exploring the Impact of 1,8-Diioodoctane on the Photostability of Organic Photovoltaics
Source: ACS Appl Energy Mater. 2024 Sep 13;7(19):8401–11. doi: 10.1021/acsaem.4c01272 (PMC11480932; doi:10.1021/acsaem.4c01272)
Supplement: Supplementary file 1 — ae4c01272_si_001.pdf [file ae4c01272_si_001.pdf]

Supporting Information

Exploring the Impact of 1,8-Diioodoctane on the Photostability of Organic Photovoltaics

Rachel C. Kilbride[a,b†], Emma L.K. Spooner[b,c,†]*, Elena J. Cassella[b], Mary E. O'Kane[b], Khalid Doudin[a], David G. Lidzey[b], Richard Jones[d] and Andrew J. Parnell[b]**

a) Department of Chemistry, The University of Sheffield, Dainton Building, Brook Hill, Sheffield, S3 7HF, United Kingdom
b) Department of Physics and Astronomy, The University of Sheffield, Hicks Building, Hounsfield Road, Sheffield, S3 7RH, United Kingdom
c) The Photon Science Institute, The University of Manchester, Oxford Road, Manchester, M13 9PY, United Kingdom
d) Department of Materials, The University of Manchester, Sackville Street Building Manchester, M1 3BB, United Kingdom

*Corresponding authors: r.c.kilbride@sheffield.ac.uk, emma.spooner@manchester.ac.uk, a.j.parnell@sheffield.ac.uk

[†]These authors contributed equally to this work

Contents

Initial Devices 2
Device Stability 6
UV-Vis Absorption 10
Photographs of Irradiated Films 12
Spectroscopic Ellipsometry 13
1H NMR 13
GIWAXS 17
AFM 22

Initial Devices

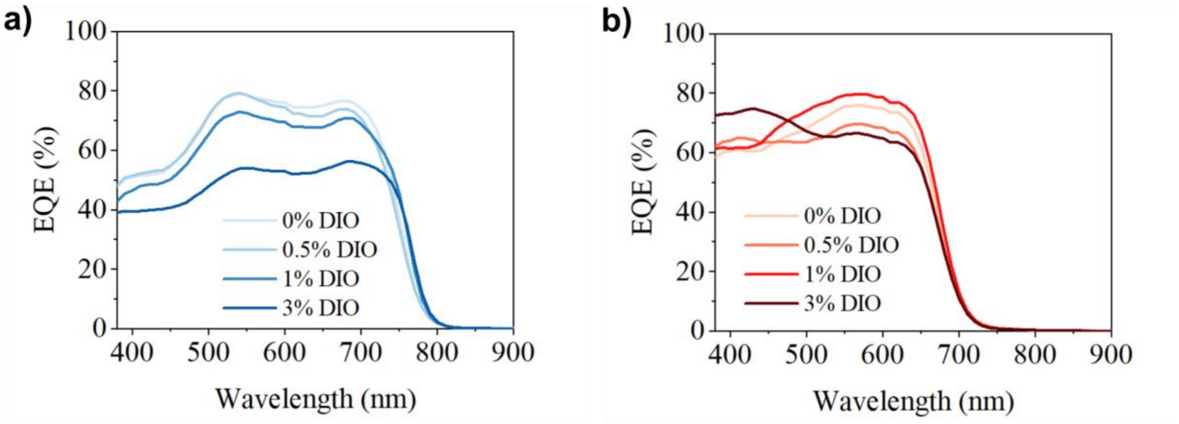

Figure S1. EQE curves for **a)** PBDB-T:ITIC and **b)** PBDB-T:$PC_{71}BM$ based devices.

Table S1. Integrated EQE values in comparison to those given by initial testing under a solar simulator.

Active Layer	DIO Content [%]	J_{SC} of initial device [mA/cm^2]	J_{SC} from integrated EQE [mA/cm^2]
PBDB-T:ITIC	0	17.1	16.5
	0.5	17.1	16.0
	1	16.2	15.4
	3	12.5	12.3
PBDB-T:$PC_{71}BM$	0	13.5	12.6
	0.5	12.8	11.8
	1	13.5	13.3
	3	13.0	12.0

Table S2. Device metrics for un-encapsulated PBDB-T:ITIC and PBDB-T:$PC_{71}BM$ cells. An average is given for 10 devices ± 1 standard deviation, with the champion value given in brackets.

Blend System	DIO Concentration [Vol%]	J_{sc} [mA cm^{-2}]	V_{oc} [V]	FF [%]	PCE [%]
PBDB-T:ITIC	0	17.5 ± 0.17 (**17.7**)	0.90 ± 0.00 (**0.91**)	65.6 ± 0.49 (**66.9**)	10.3 ± 0.12 (**10.5**)
	0.5	17.3 ± 0.23 (**17.7**)	0.89 ± 0.00 (**0.90**)	66.6 ± 1.23 (**68.4**)	10.1 ± 0.35 (**10.7**)
	1	16.7 ± 0.34 (**17.1**)	0.89 ± 0.01 (**0.90**)	56.1 ± 2.18 (**58.8**)	8.19 ± 0.39 (**8.67**)
	3	11.5 ± 0.15 (**11.6**)	0.82 ± 0.06 (**0.87**)	49.8 ± 3.76 (**54.0**)	4.64 ± 0.72 (**5.30**)
PBDB-T:$PC_{71}BM$	0	13.0 ± 0.25 (**13.3**)	0.88 ± 0.00 (**0.89**)	66.6 ± 0.39 (**67.5**)	7.55 ± 0.16 (**7.83**)
	0.5	13.6 ± 0.14 (**13.8**)	0.87 ± 0.00 (**0.88**)	68.3 ± 0.32 (**68.8**)	8.07 ± 0.15 (**8.31**)
	1	12.9 ± 0.13 (**13.2**)	0.86 ± 0.00 (**0.87**)	65.7 ± 0.40 (**66.5**)	7.29 ± 0.10 (**7.50**)
	3	14.2 ± 0.21 (**14.6**)	0.86 ± 0.00 (**0.87**)	71.1 ± 0.57 (**72.3**)	8.60 ± 0.22 (**9.04**)

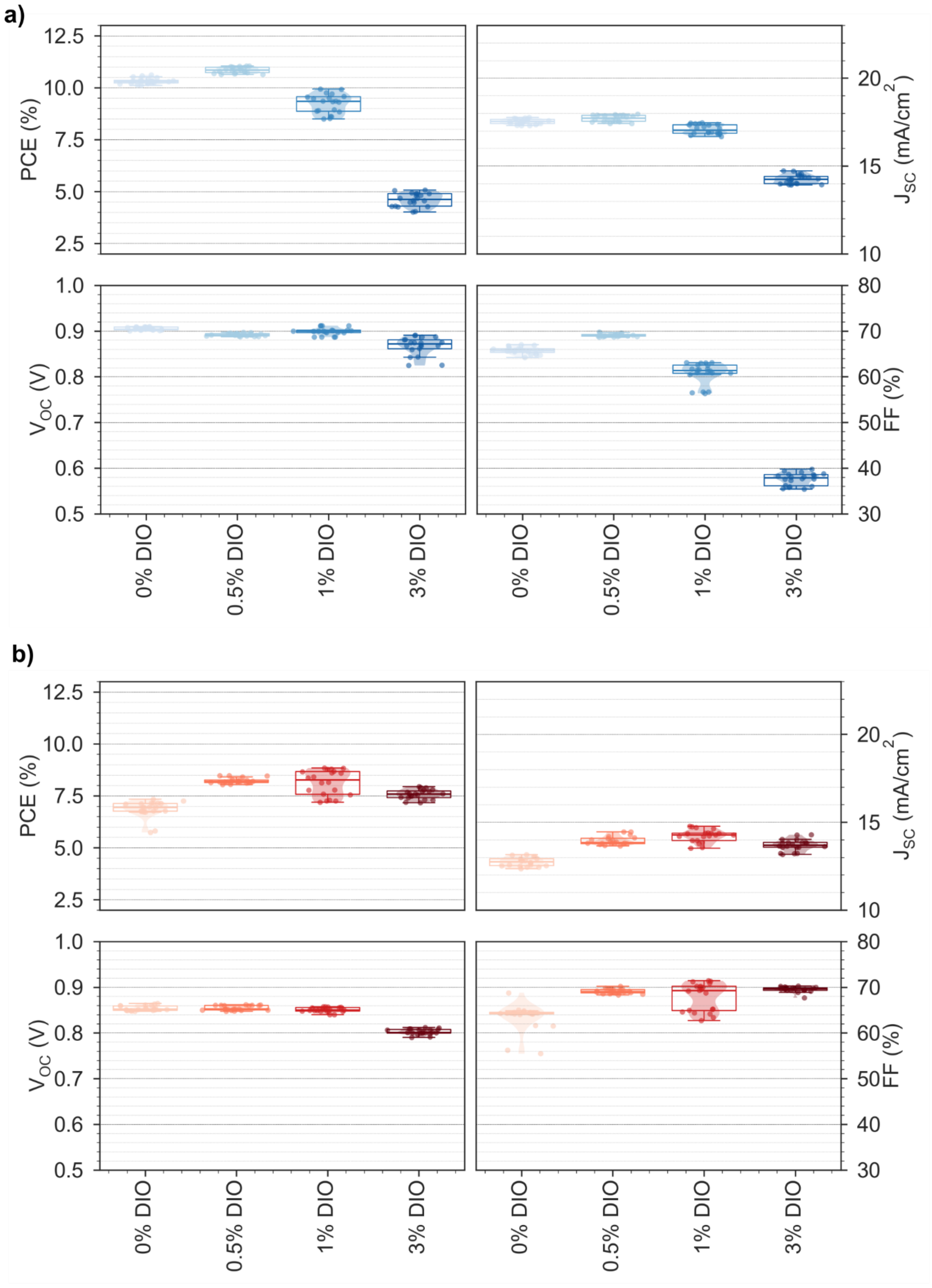

Figure S2. A boxplot showing the statistical relationship between device performance for **a)** PBDB-T:ITIC based devices, and **b)** PBDB-T:$PC_{71}BM$ based, encapsulated devices.

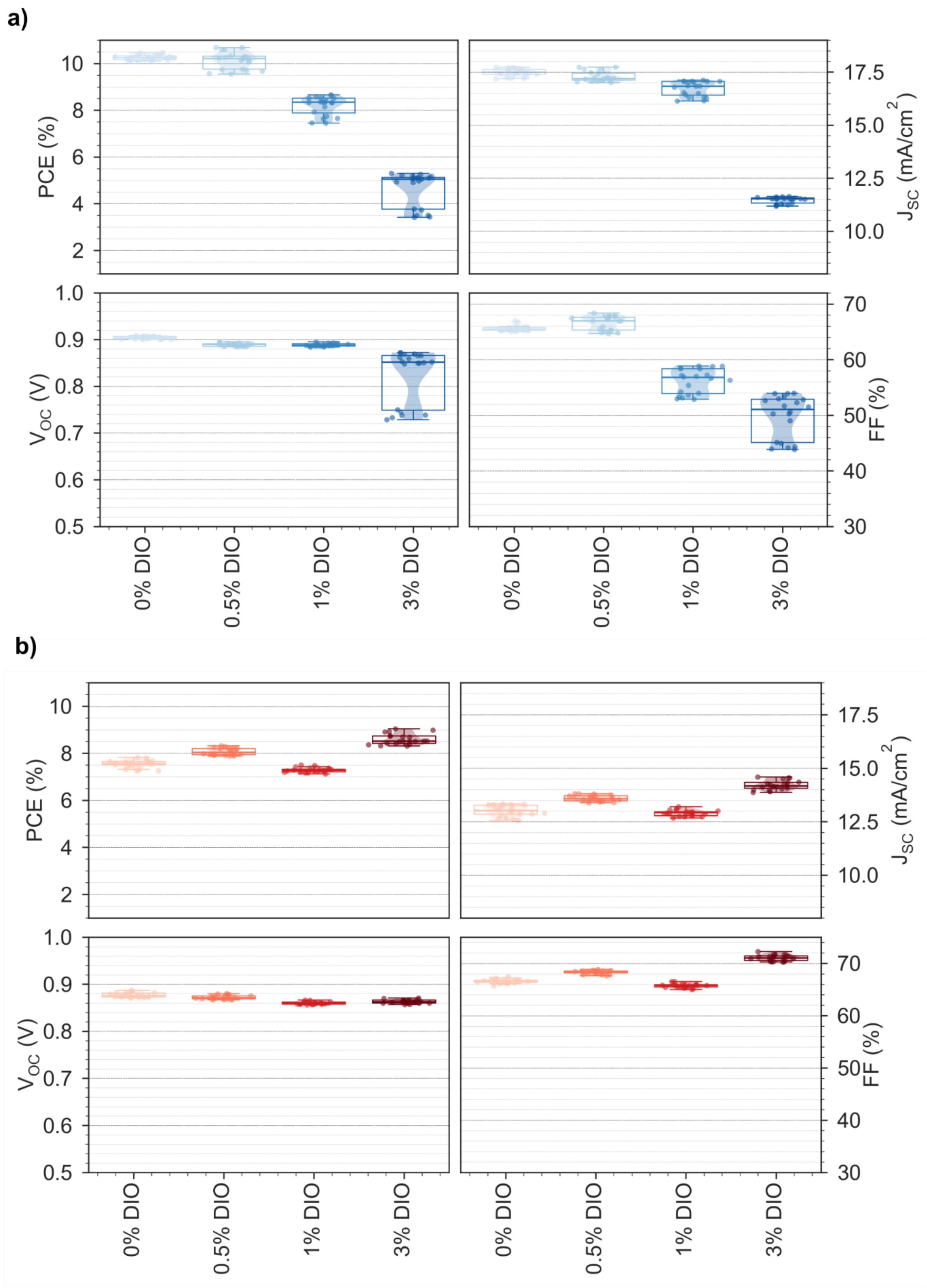

Figure S3. A boxplot showing the statistical relationship between device performance for **a)** PBDB-T:ITIC based devices, and **b)** PBDB-T:$PC_{71}BM$ based, un-encapsulated devices.

Device Stability

T_{80} lifetime values for OPV devices aged under different conditions, with varying DIO content. In all cases the T_{80} refers to the time taken to reach 80% of the initial device performance. If not reached during the testing time, the T_{80} was extrapolated via fitting of the data.

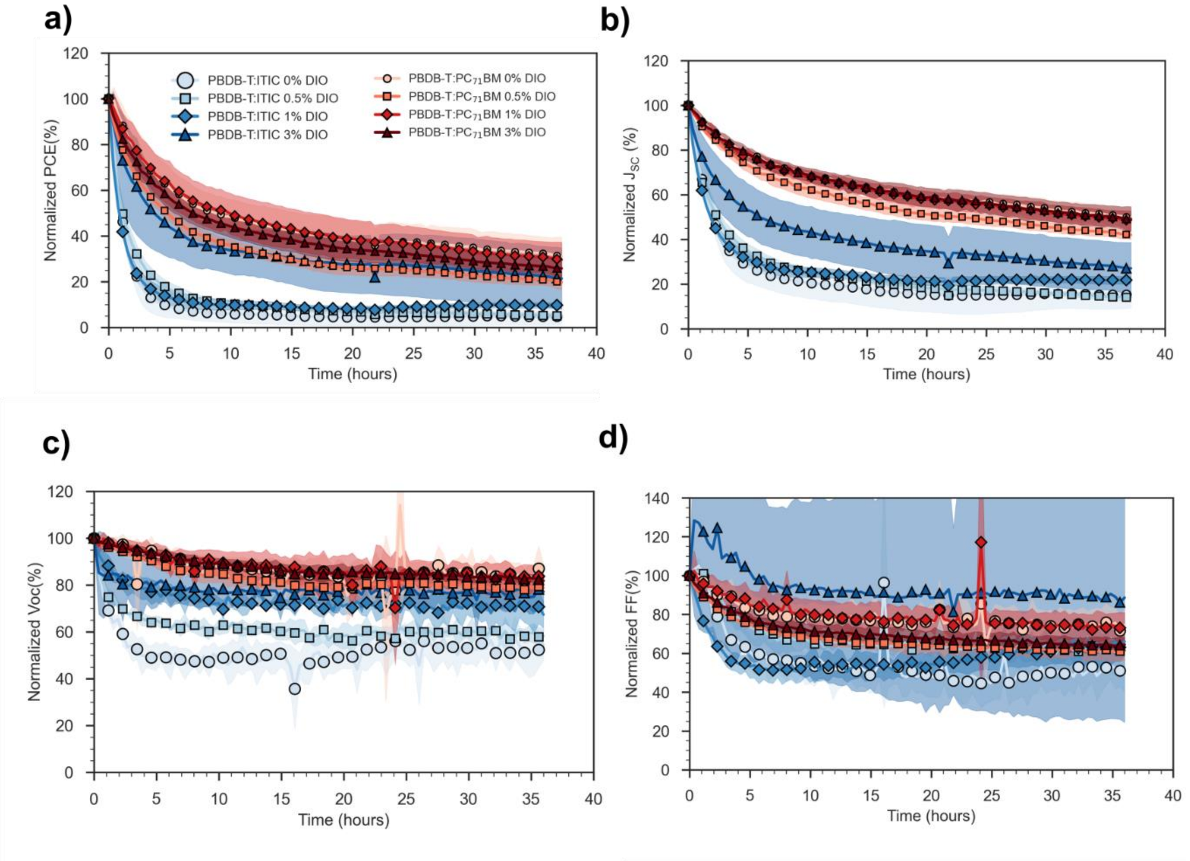

Figure S4. Normalized PV device parameters (a) *PCE* (b) J_{sc}, (c) V_{oc} and (d) FF of PBDB-T:ITIC and PBDB-T:PC_{71}BM based un-encapsulated devices, processed with 0-3 Vol% DIO during exposure to 1 Sun simulated solar irradiation under ambient conditions, at V_{oc} (in an ATLAS lifetime tester). In all cases 4 pixels are averaged across two different substrates. To reduce the impact of noise on the clarity of the data, points are plotted every 3 measurements.

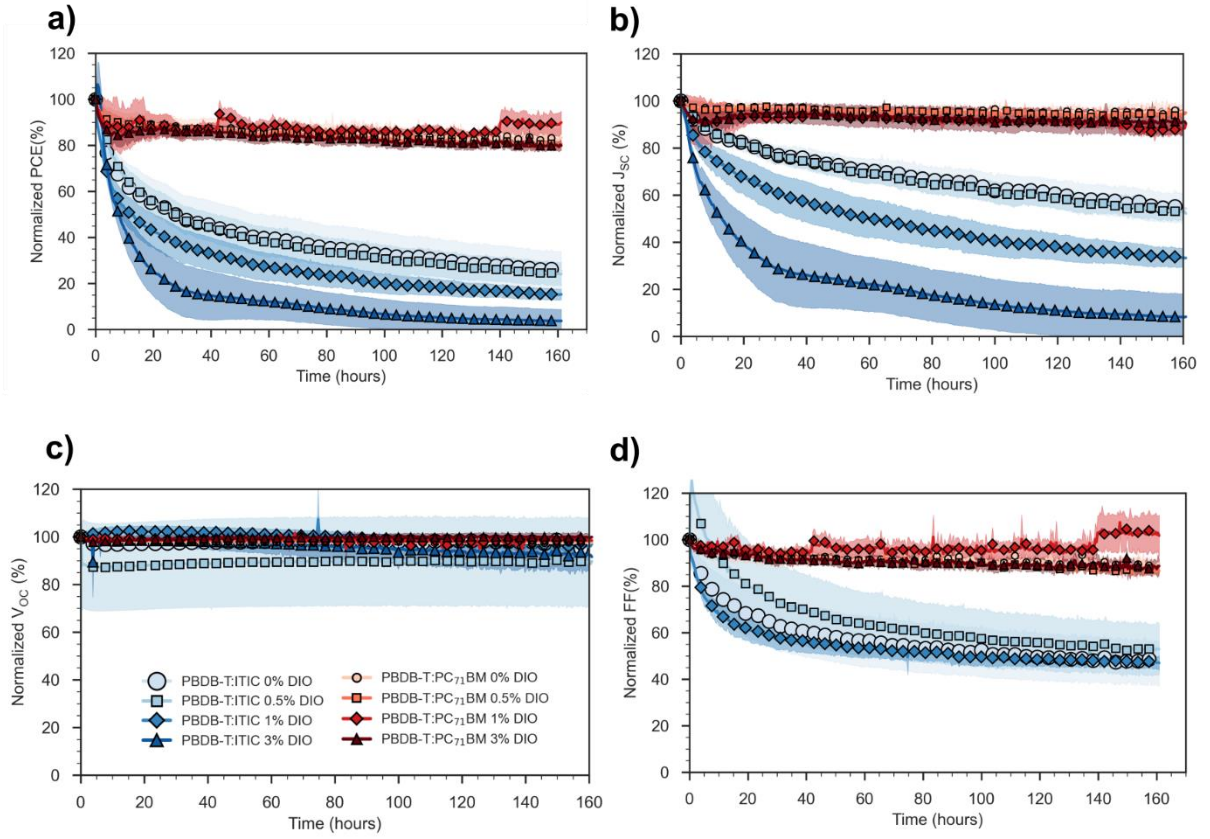

Figure S5. Normalized PV device parameters **(a)** *PCE* **(b)** J_{sc}, **(c)** V_{oc} and **(d)** FF of PBDB-T:ITIC and PBDB-T:PC$_{71}$BM based encapsulated devices, processed with 0-3 Vol% DIO during exposure to 1 Sun simulated solar irradiation under ambient conditions, at V_{oc} (in an ATLAS lifetime tester). To reduce the impact of noise on the clarity of the data, points are plotted every 10 measurements.

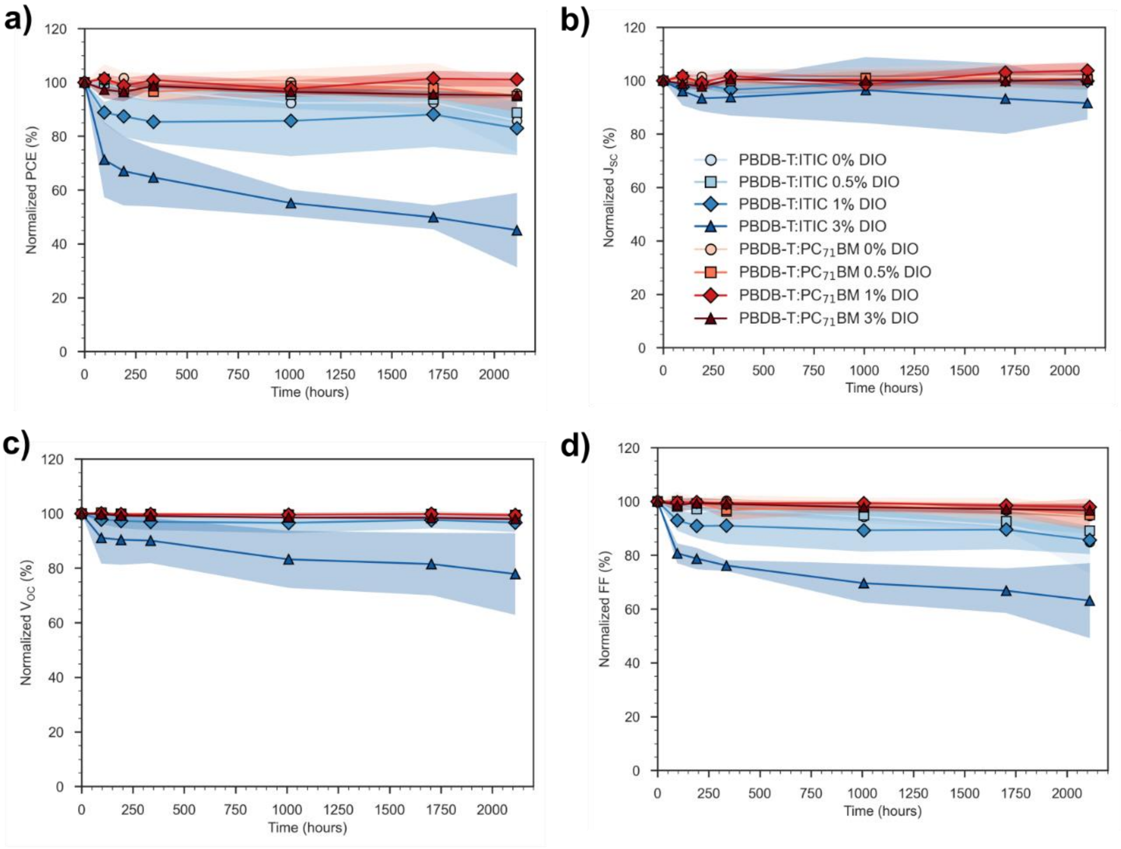

Figure S6. Normalized PV device parameters **(a)** PCE **(b)** J_{sc}, **(c)** V_{oc} and **(d)** FF of PBDB-T:ITIC and PBDB-T:$PC_{71}BM$ based encapsulated devices, processed with 0-3 Vol% DIO, during storage in the dark under ambient conditions. In all cases at least 2 pixels are averaged.

Table S3. T_{80} lifetime values for OPV devices aged under different conditions, with varying DIO content. In all cases the T_{80} refers to the time taken to reach 80% of the initial device performance. If not reached during the testing time, the T_{80} was extrapolated via fitting of the data.

Active Layer	DIO Content [%]	Ageing Conditions	T_{80} [hours]
PBDB-T:ITIC	0	Un-encapsulated devices under 1 Sun, ambient conditions, V_{oc}	0.3
	0.5		0.3
	1		0.2
	3		0.9
PBDB-T:$PC_{71}BM$	0		1.9
	0.5		1.1
	1		2.0
	3		1.5
PBDB-T:ITIC	0	Encapsulated devices under 1 Sun, ambient conditions, V_{oc}	3.4
	0.5		4.9
	1		2.1
	3		2.5
PBDB-T:$PC_{71}BM$	0		192
	0.5		147
	1		642
	3		149
PBDB-T:ITIC	0	Encapsulated devices kept in the dark, ambient conditions	3378
	0.5		4427
	1		3091
	3		67
PBDB-T:$PC_{71}BM$	0		10920
	0.5		11841
	1		106650
	3		11451

UV-Vis Absorption

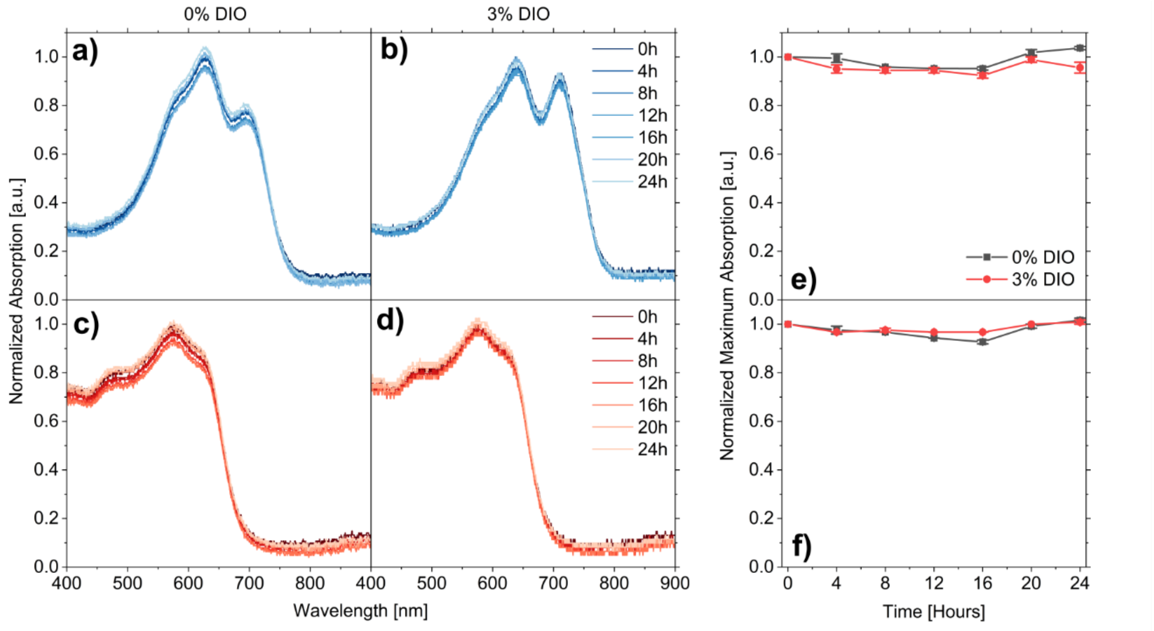

Figure S7. Normalized UV-Vis absorption of **(a, b)** PBDB-T:ITIC and (**c, d**) PBDB-T:$PC_{71}BM$ blend films processed with 0% and 3% DIO during storage in the dark under ambient conditions. Corresponding normalized maximum absorption as a function of ageing time for **(e)** PBDB-T:ITIC and **(f)** PBDB-T:$PC_{71}BM$ blend films. Data are normalized to the initial measurement at 0h at the wavelength corresponding to the maximum absorption (626 nm and 638 nm for PBDB-T:ITIC and 576 nm and 570 nm for PBDB-T:$PC_{71}BM$ blend films processed with 0 and 3 Vol% DIO respectively).

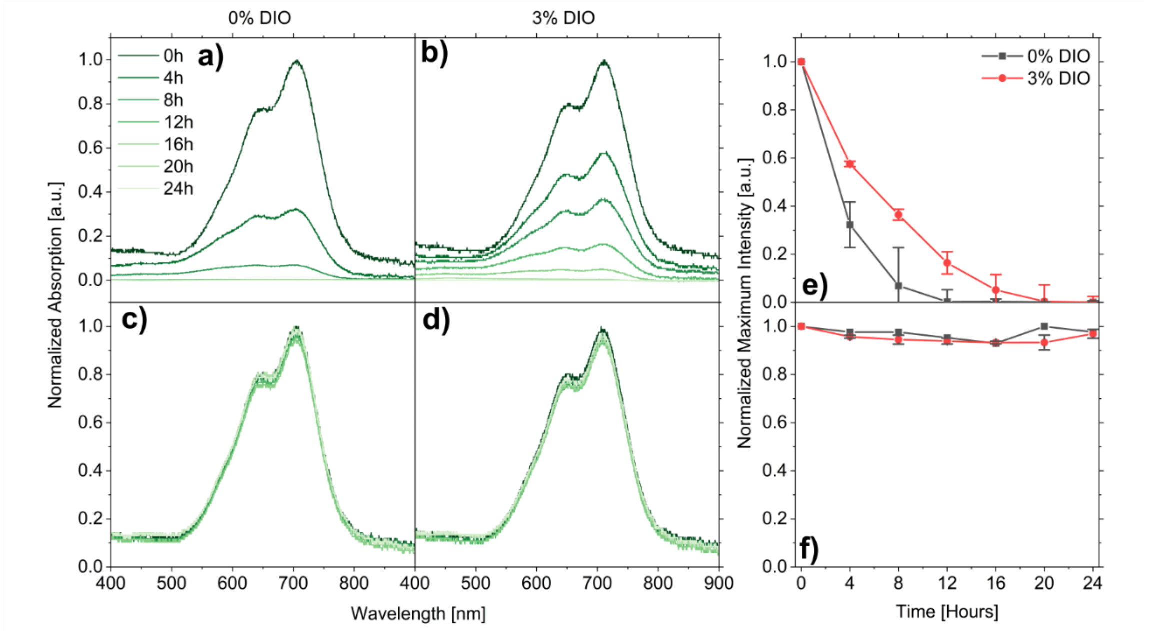

Figure S8. Normalized UV-Vis absorption of neat ITIC films processed with 0% and 3% DIO during **(a, b)** exposure to 1 Sun simulated solar irradiation under ambient conditions and **(c, d)** storage in the dark under ambient conditions. Corresponding normalized maximum absorption as a function of ageing time during **(e)** exposure to 1 Sun simulated solar irradiation under ambient conditions and **(f)** storage in the dark under ambient conditions. Data are normalized to the initial measurement at 0 h at the wavelength corresponding to the maximum absorption (706 nm and 710 nm for films processed with 0 and 3 Vol% DIO respectively).

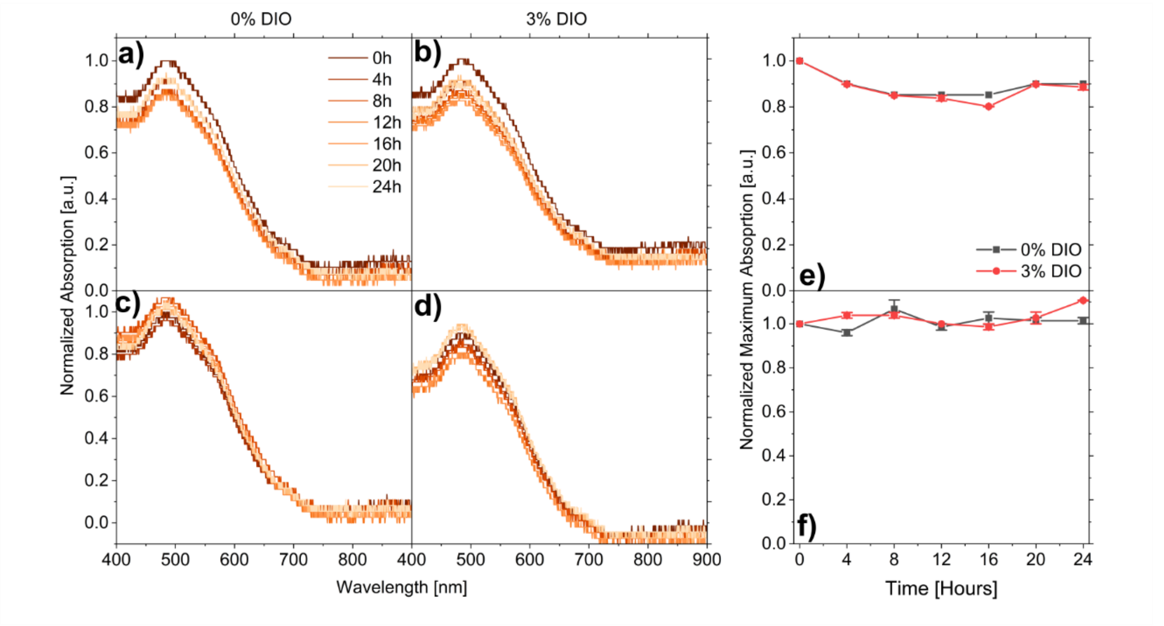

Figure S9. Normalized UV-Vis absorption of neat $PC_{71}BM$ films processed with 0% and 3% DIO during **(a ,b)** exposure to 1 Sun simulated solar irradiation under ambient conditions and **(c, d)** storage in the dark under ambient conditions. Corresponding normalized maximum absorption as a function of ageing time during **(e)** exposure to 1 Sun simulated solar irradiation under ambient conditions and **(f)** storage in the dark under ambient conditions. Data are normalized to the initial measurement at 0 h at the wavelength corresponding to the maximum absorption (475 nm and 478 nm for films processed with 0 and 3 Vol% DIO respectively).

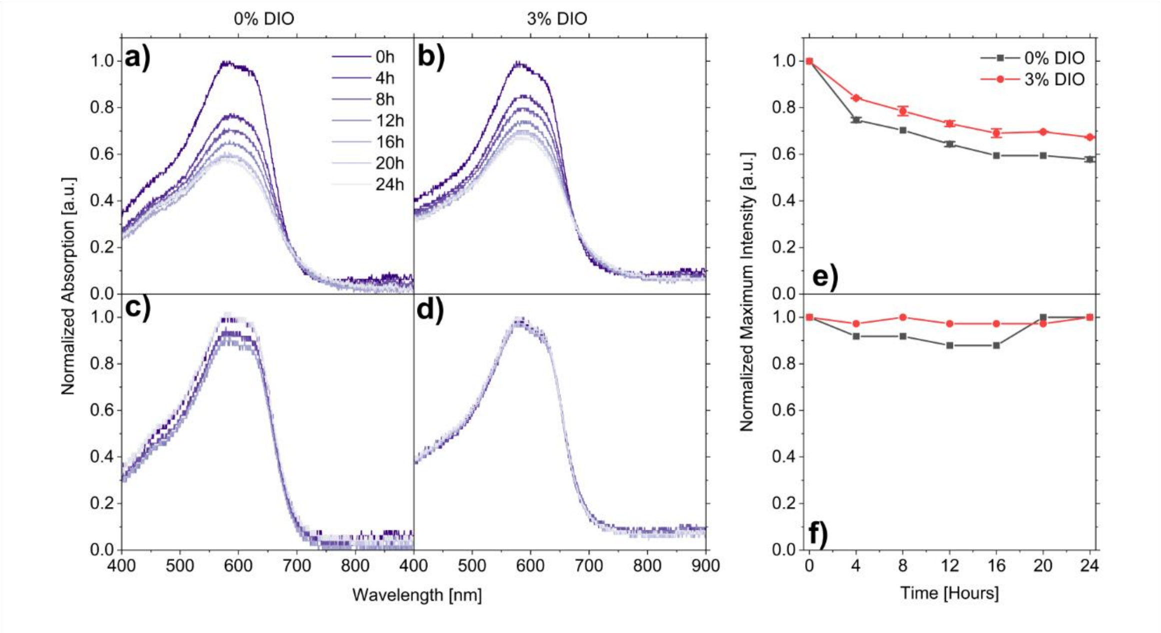

Figure S10. Normalized UV-Vis absorption of neat PBDB-T films processed with 0% and 3% DIO during **(a, b)** exposure to 1 Sun simulated solar irradiation under ambient conditions and **(c, d)** storage in the dark under ambient conditions. Corresponding normalized maximum absorption as a function of ageing time during **(e)** exposure to 1 Sun simulated solar irradiation under ambient conditions and **(f)** storage in the dark under ambient conditions. Data are normalized to the initial measurement at 0 h at the wavelength corresponding to the maximum absorption (573 nm and 576 nm for films processed with 0 and 3 Vol% DIO respectively).

Photographs of Irradiated Films

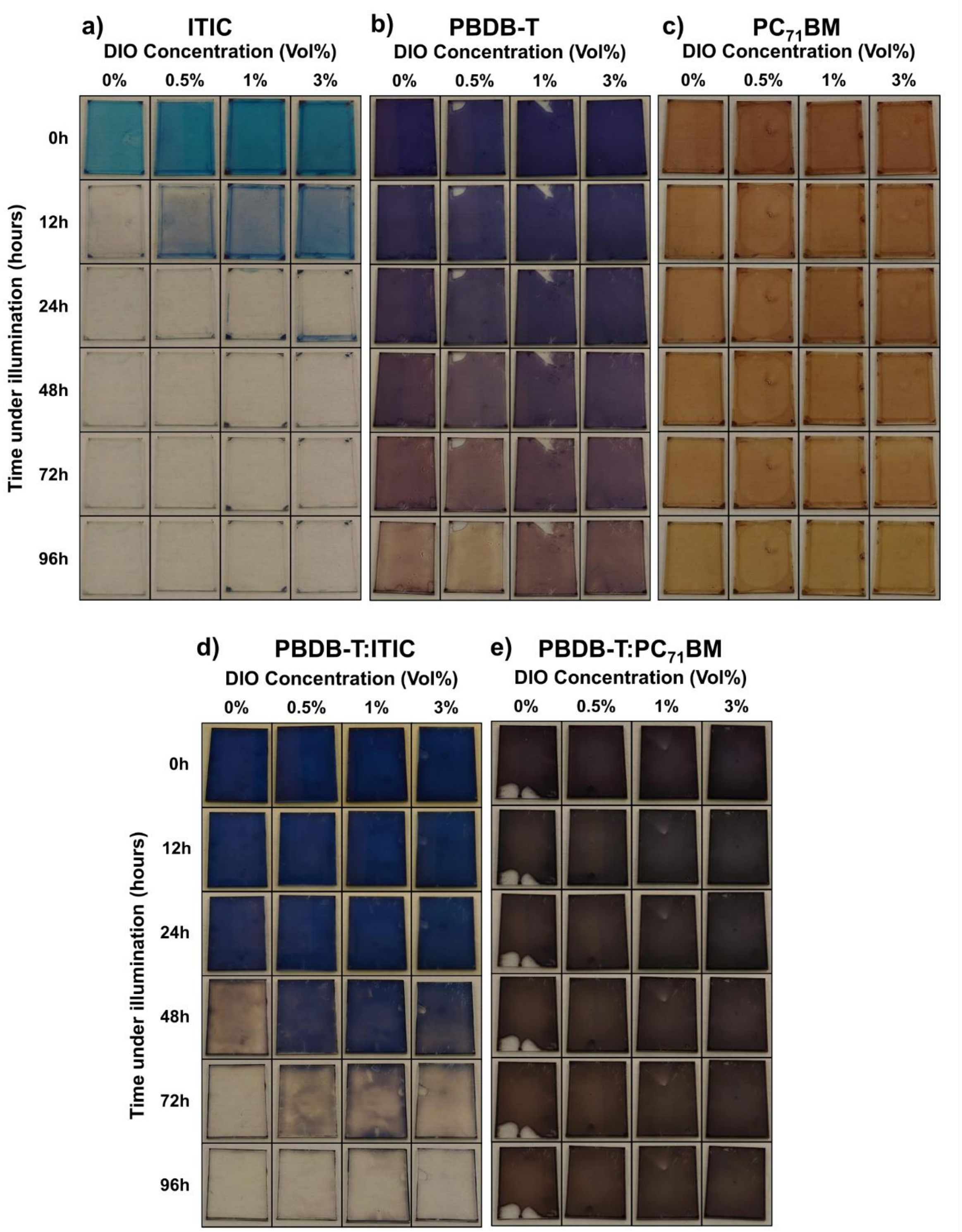

Figure S11. Photographs of **(a)** ITIC, **(b)** PBDB-T, **(c)** $PC_{71}BM$, **(d)** PBDB-T:ITIC and **(e)** PBDB-T:$PC_{71}BM$ films processed with 0-3 Vol% DIO and aged under 1 Sun irradiation for a total duration of 96h with photographs taken every 12h up to 48h then every 24h.

Spectroscopic Ellipsometry

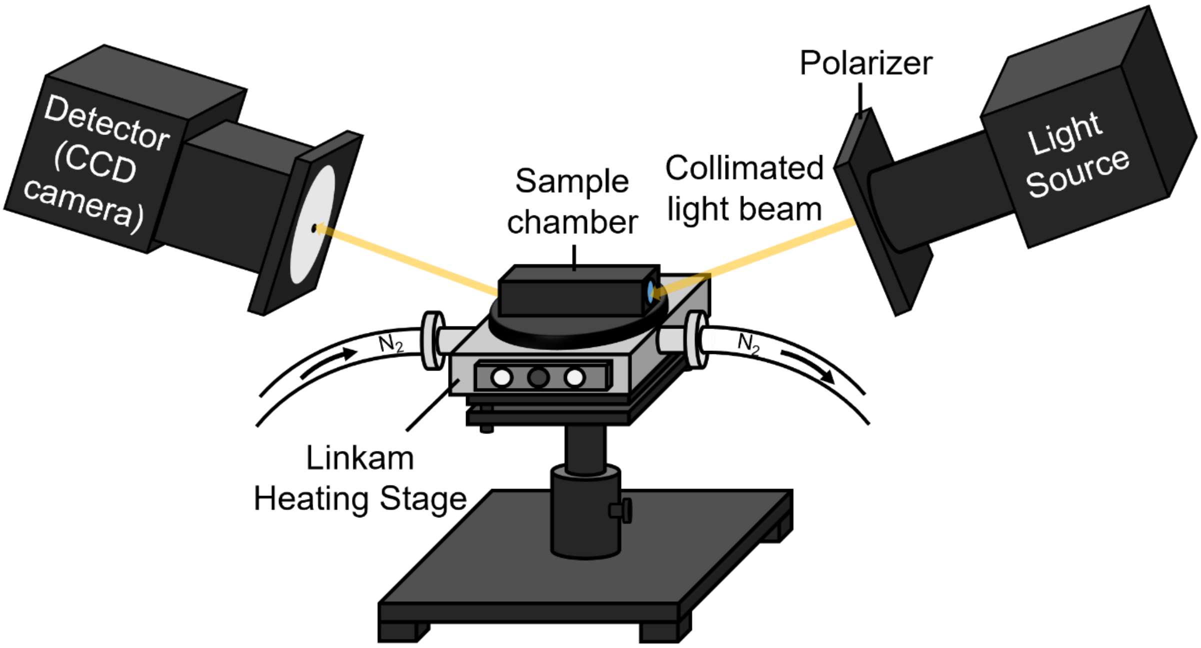

Figure S12. Spectroscopic *in-situ* ellipsometry setup for the isothermal annealing of thin films. The sample is mounted on a Linkam heating stage and enclosed in a chamber with continuous nitrogen flow.

1H NMR

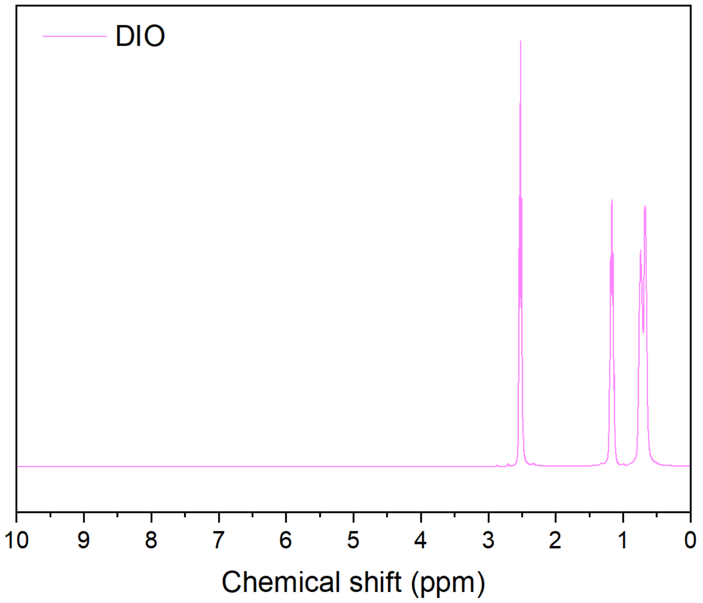

Figure S13. ^{1}H NMR of DIO

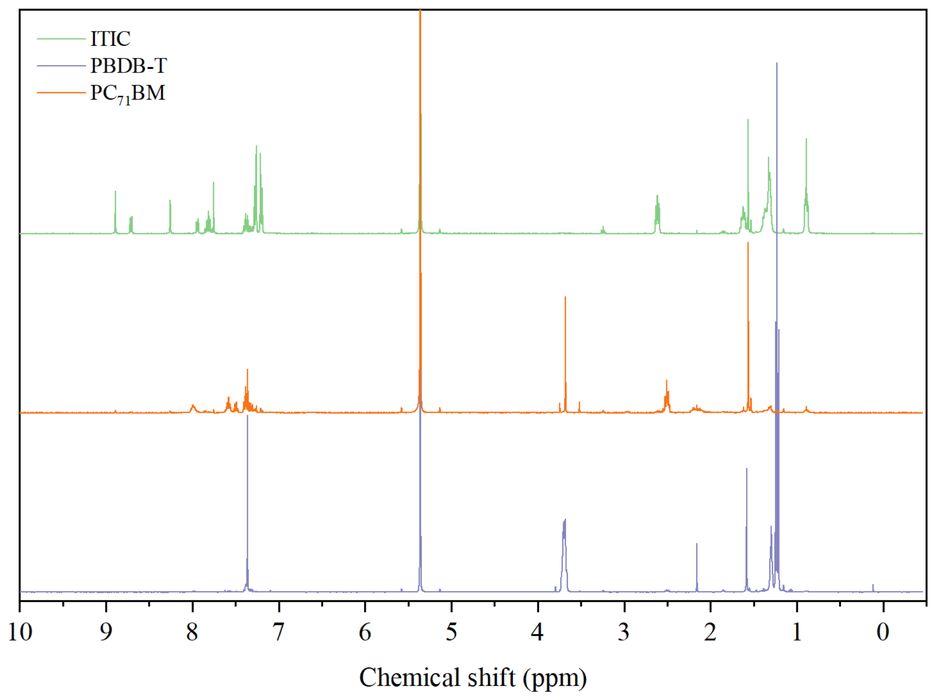

Figure S14. ^{1}H NMR of pure ITIC, $PC_{71}BM$, PBDB-T. Curves vertically offset for clarity.

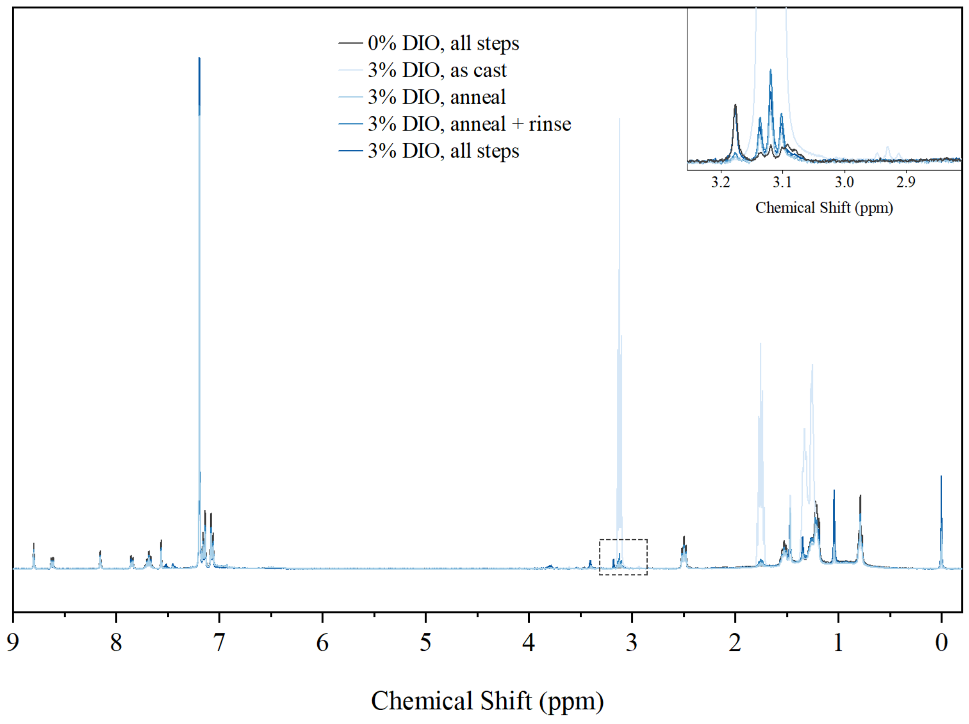

Figure S15. Full ^{1}H NMR spectra of PBDB-T:ITIC films after various processing steps. Box represents the area shown in the inset (replicated in the main text **Figure 4c**).

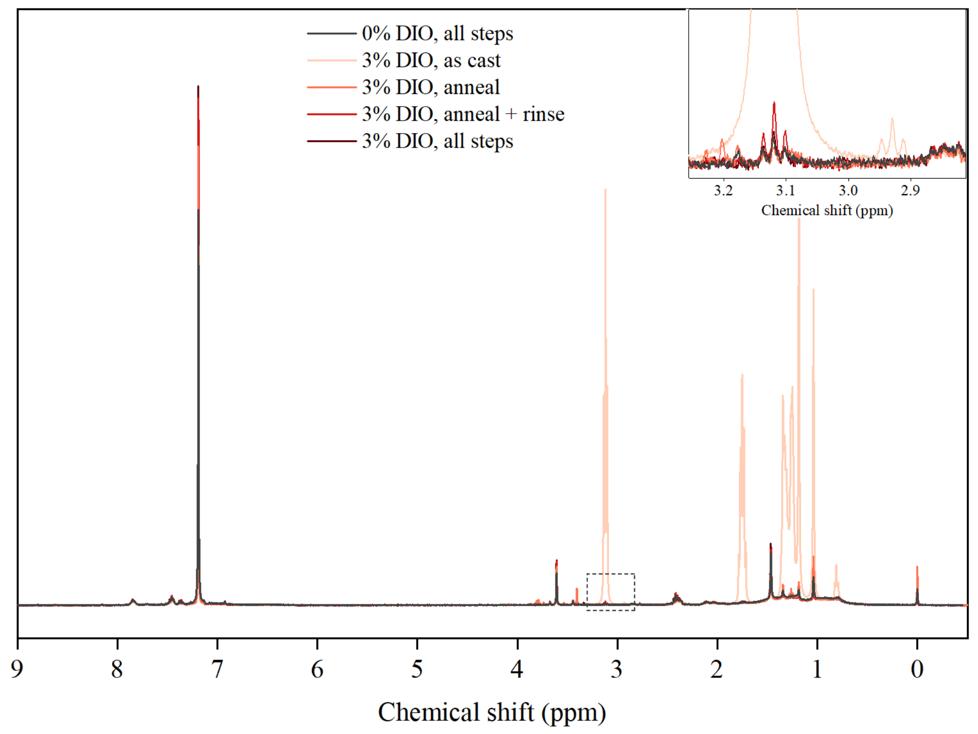

Figure S16. Full ^{1}H NMR spectra of PBDB-T:$PC_{71}BM$ films after various processing steps. Box represents the area shown in the inset (replicated in the main text Fig. 4d).

Supplementary Note 1

Here we use the ratio of peak integration areas of DIO and each acceptor in ^{1}H NMR to qualitatively compare DIO content between samples, following the procedure set out by Jacobs *et al.*[2] In the latter work PBDB-T is used to compare samples, however here there were no peaks well separated enough from the donor peaks within the blend to use this technique, so the acceptor peaks are used instead. The identifying peaks for each acceptor are shown below, in **Figure S16**. Jacobs' work notes that any well-separated, unique peak can be used, and the ratio will be the same:

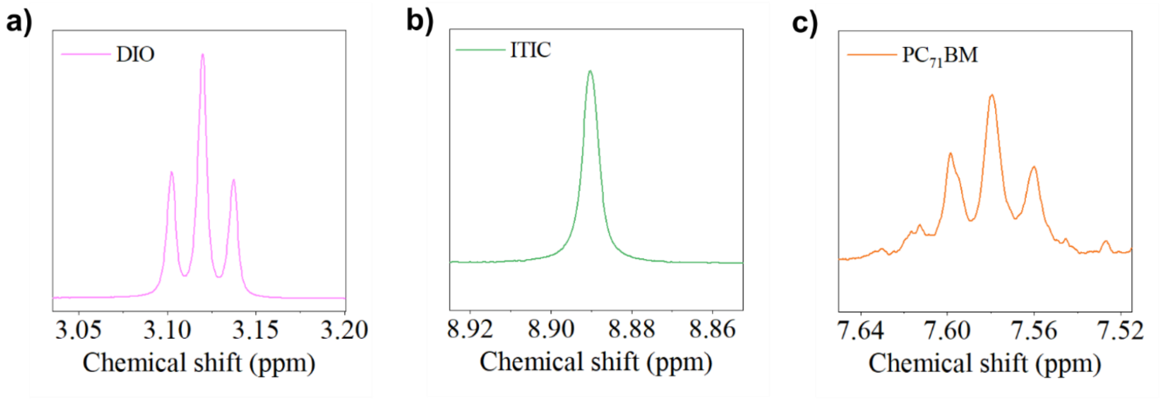

Figure S17. Identifying peaks of each component that were integrated to compare DIO content between samples. **a)** DIO, **b)** ITIC and **c)** $PC_{71}BM$.

As peak integration area is considered proportional to molar concentration, we considered the differences between ITIC and $PC_{71}BM$ to provide a correction factor. The PBDB-T:$PC_{71}BM$ films are prepared at a lower solid concentration than that of PBDB-T:ITIC (15 mg/mL compared to 18 mg/mL), as this is optimized for device performance. This means the films will contain less mass of $PC_{71}BM$, if it is assumed that the same volume of ink forms the wet film. The molecular weights of ITIC and $PC_{71}BM$ are also different (1423 g/mol compared to 1031 g/mol), and as a result their moles from equivalent mass. These differences in molar concentration can be summarized as:

$$\frac{ITIC\,\frac{\mathrm{mol}}{\mathrm{mL}}}{PCBM\,\frac{\mathrm{mol}}{\mathrm{mL}}} = \frac{\left(\frac{0.009\,\frac{\mathrm{g}}{\mathrm{mL}}}{1423\,\frac{\mathrm{g}}{\mathrm{mol}}}\right)}{\left(\frac{0.0075\,\frac{\mathrm{g}}{\mathrm{mL}}}{1031\,\frac{\mathrm{g}}{\mathrm{mol}}}\right)} = 0.87$$

Here, the mass concentration used is half that of the solution as all inks are made in a 1:1 donor:acceptor ratio. From this relation, we know in a given volume there are less moles of ITIC than $PC_{71}BM$. This means that the same moles of DIO in a solution will yield a higher DIO:Acceptor ratio for ITIC than $PC_{71}BM$. As a result, all ratios obtained for $PC_{71}BM$ films are corrected to obtain moles of DIO that can be compared to the ITIC films:

$$moles\ DIO{:}ITIC = \frac{moles\ DIO{:}PCBM}{0.87}$$

The results of the corrected integrations can be seen in the main text and are summarized below.

Table S4. Corrected DIO:Acceptor molar concentrations.

**Conditions**	**DIO:Acceptor for PBDB-T:ITIC**	**DIO:Acceptor for PBDB-T:$PC_{71}BM$**
0% DIO, all steps	0.57	0.15
3% DIO, as cast	43.5	43.0
3% DIO, annealed	1.22	0.16
3% DIO, annealed + rinse	1.61	0.25
3% DIO, all steps	1.37	0.14

Support for the validity of this method can be found in the very similar ratios for the two blends with 3% DIO and no other processing steps (as-cast). Here an identical volume will have been used to form each film, and the same number of films used to fabricate the NMR solution, and as such the moles of DIO should be identical.

GIWAXS
Fresh Samples

ITIC

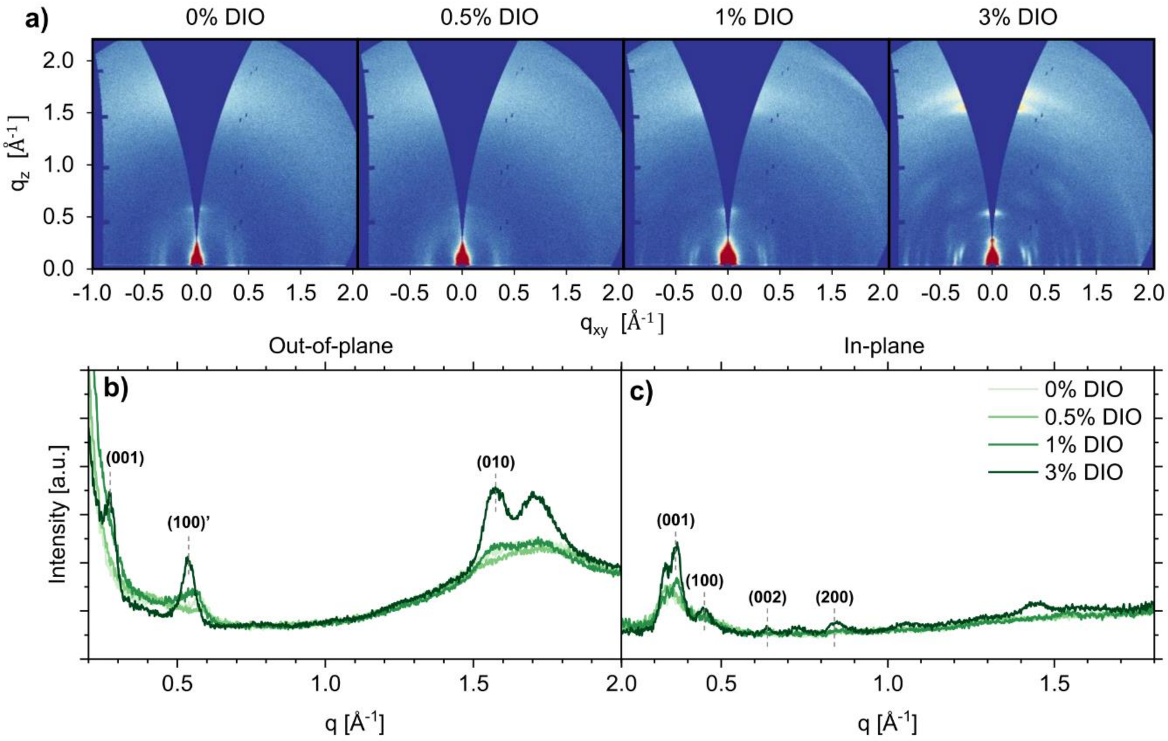

Figure S18. (a) 2D GIWAXS patterns of neat ITIC films processed with 0, 0.5, 1 and 3 Vol% DIO. Corresponding **(b)** out-of-plane and **(c)** in-plane 1D azimuthally integrated intensity profiles. Peaks indexing corresponds to the ITIC bimodal lamellar stacking reported elsewhere.[3]

$PC_{71}BM$

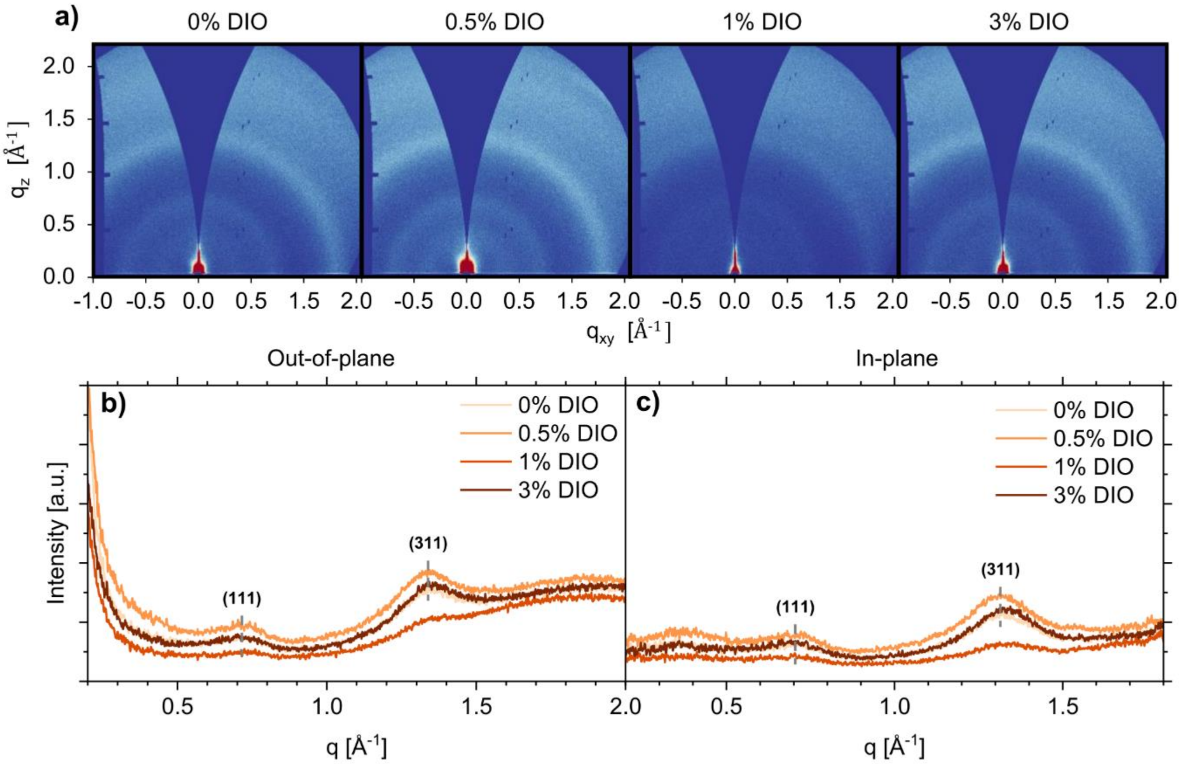

Figure S19. (a) 2D GIWAXS patterns of neat $PC_{71}BM$ films processed with 0, 0.5, 1 and 3 Vol% DIO. Corresponding **(b)** out-of-plane and **(c)** in-plane 1D azimuthally integrated intensity profiles. The two most prominent peaks are consistent with $PC_{71}BM$ GIWAXS reported elsewhere, corresponding to a hexagonal-close packed lattice of $PC_{71}BM$ aggregates.[4,5]

PBDB-T

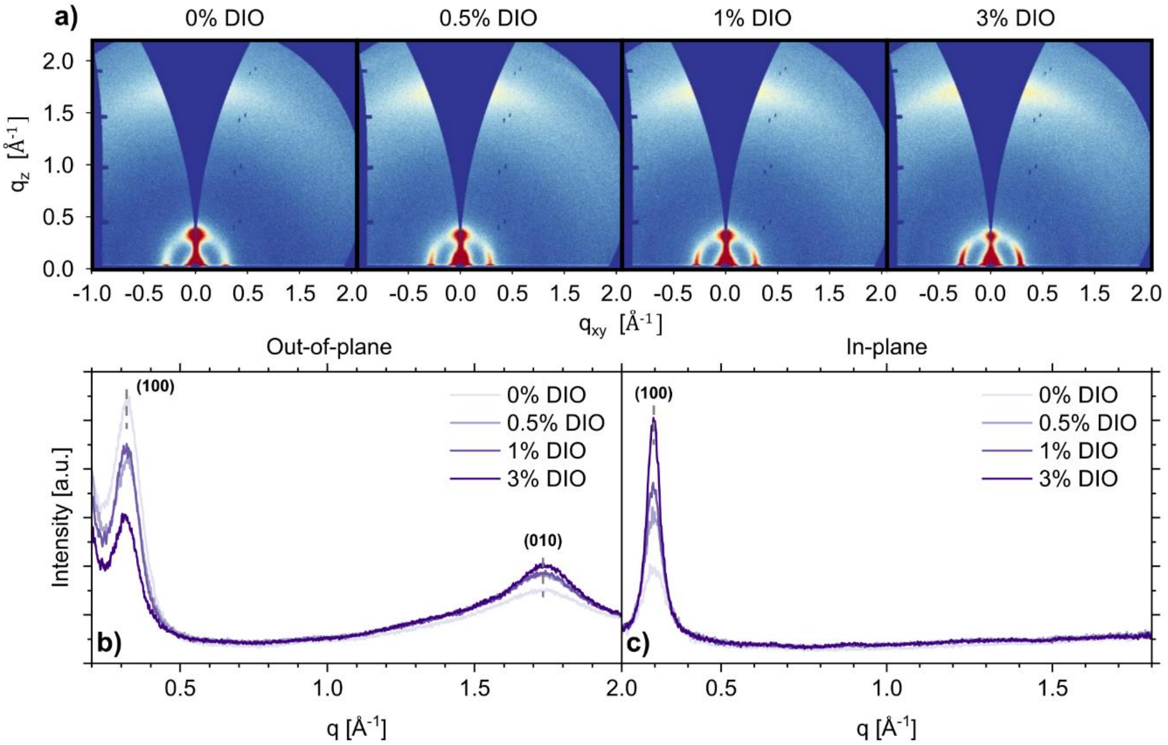

Figure S20. (a) 2D GIWAXS patterns of neat PBDB-T films processed with 0, 0.5, 1 and 3 Vol% DIO. Corresponding **(b)** out-of-plane and **(c)** in-plane 1D azimuthally integrated intensity profiles. The (100) and (010) scattering features are consistent with PBDB-T GIWAXS reported elsewhere.[3]

Angle-Dependent Intensity Profiles

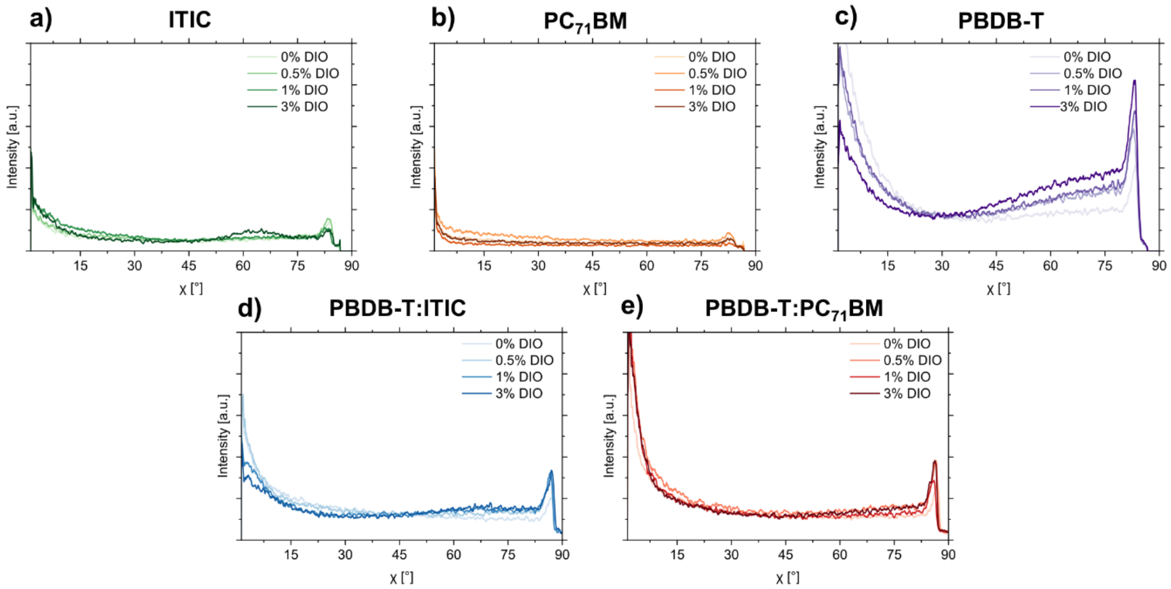

Figure S21. Azimuthal angle (χ) dependent GIWAXS intensity profiles of **(a)** ITIC, **(b)** $PC_{71}BM$, **(c)** PBDB-T, **(d)** PBDB-T:ITIC and **(e)** PBDB-T:$PC_{71}BM$. Integrations were performed across the full azimuthal angle range between q = 0.2 $Å^{-1}$ and q = 0.4 $Å^{-1}$. Here, χ = 0 ° corresponds to the out-of-plane direction (q_z direction in reciprocal space) and χ = 90 ° corresponds to the in-plane direction (q_{xy} direction in reciprocal space).

Aged Samples

PBDB-T:ITIC

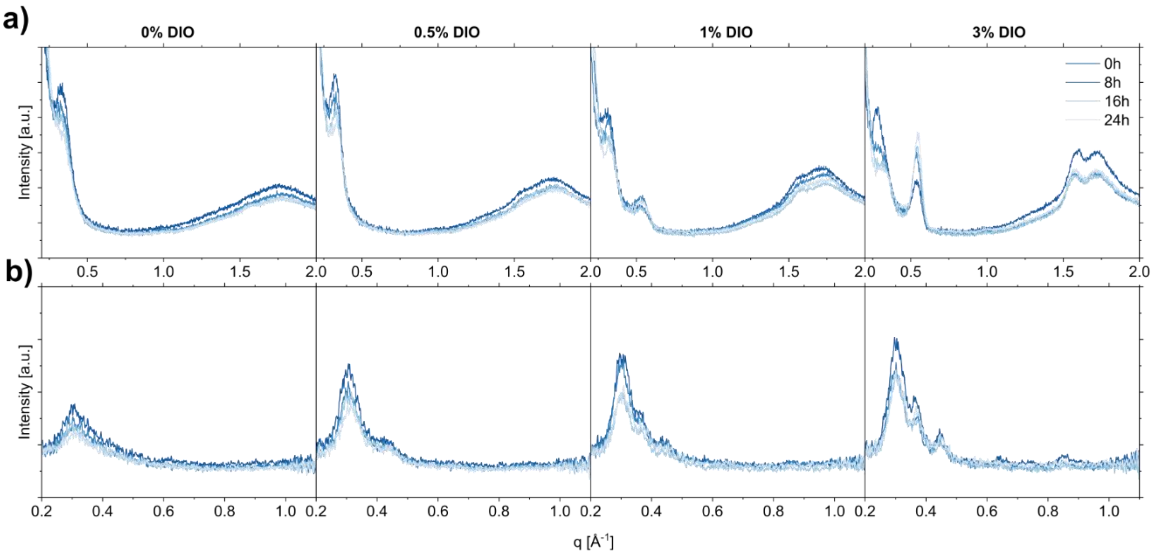

Figure S22. (a) Out-of-plane and **(b)** in-plane 1D GIWAXS intensity profiles of PBDB-T:ITIC blend films processed with 0-3Vol% DIO during 1 Sun simulated solar irradiation under ambient conditions for a total duration of 24 h with measurements taken every 8 h.

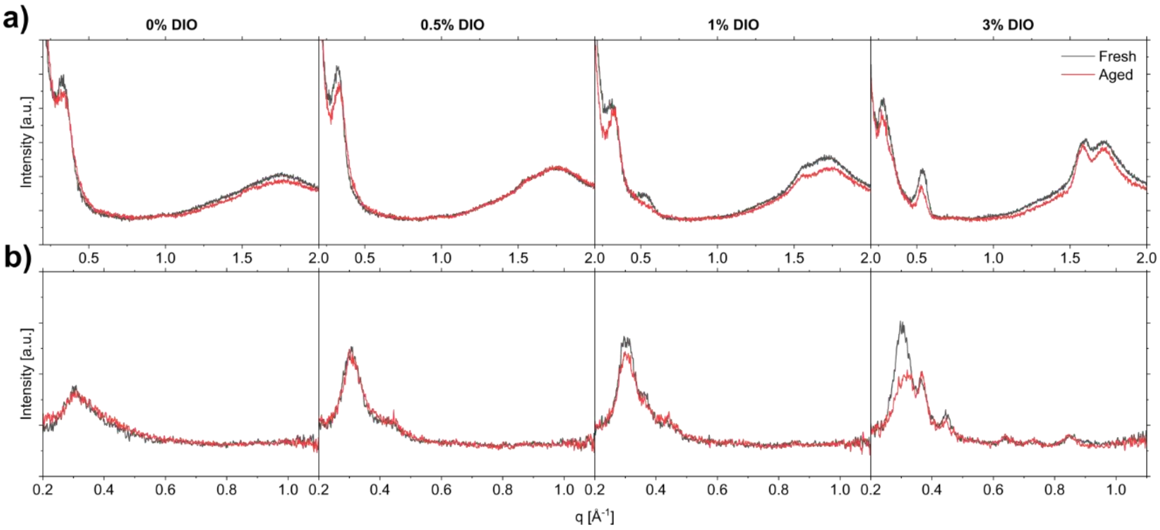

Figure S23. (a) Out-of-plane and **(b)** in-plane 1D GIWAXS intensity profiles of PBDB-T:ITIC blend films processed with 0-3Vol% DIO measured immediately after fabrication (labelled *Fresh*) and after ageing in the dark under ambient conditions for 2 weeks (labelled *Aged*).

PBDB-T:$PC_{71}BM$

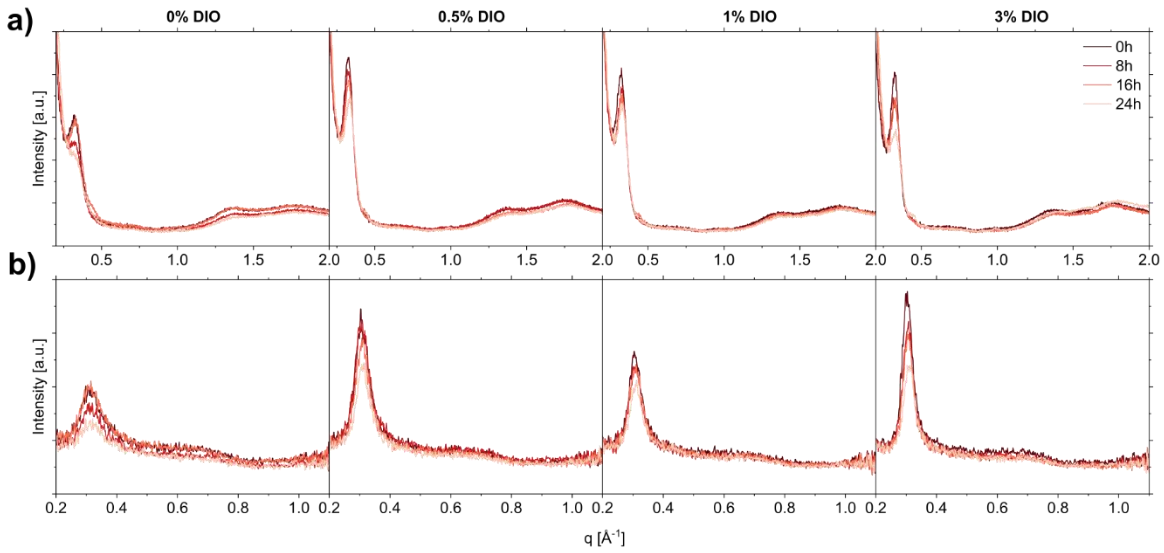

Figure S24. (a) Out-of-plane and **(b)** in-plane 1D GIWAXS intensity profiles of PBDB-T:$PC_{71}BM$ blend films processed with 0-3Vol% DIO during 1 Sun simulated solar irradiation under ambient conditions for a total duration of 24 h with measurements taken every 8 h.

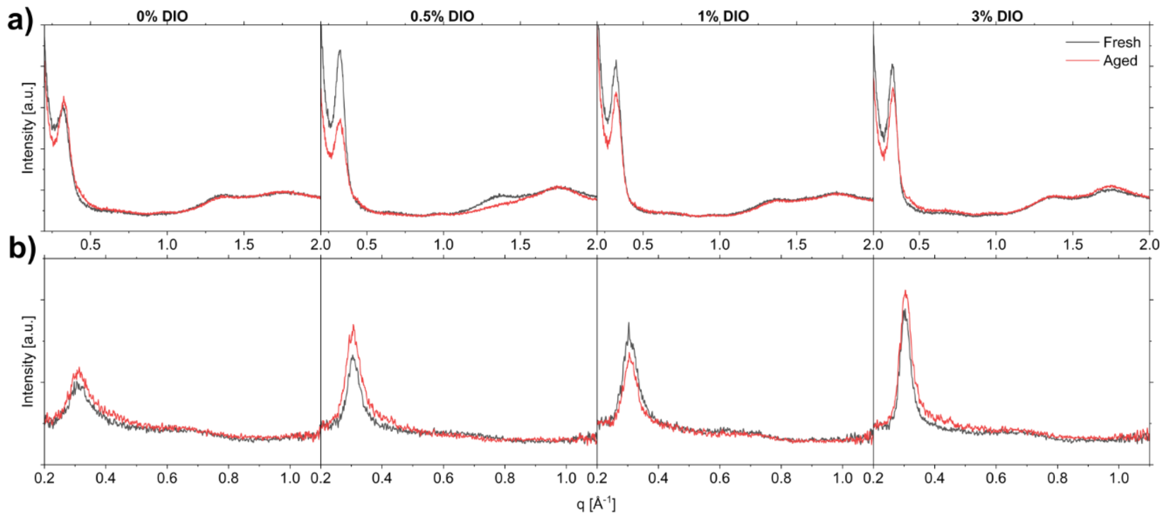

Figure S25. (a) Out-of-plane and **(b)** in-plane 1D GIWAXS intensity profiles of PBDB-T:$PC_{71}BM$ blend films processed with 0-3Vol% DIO measured immediately after fabrication (labelled *Fresh*) and after ageing in the dark under ambient conditions for 2 weeks (labelled *Aged*).

AFM

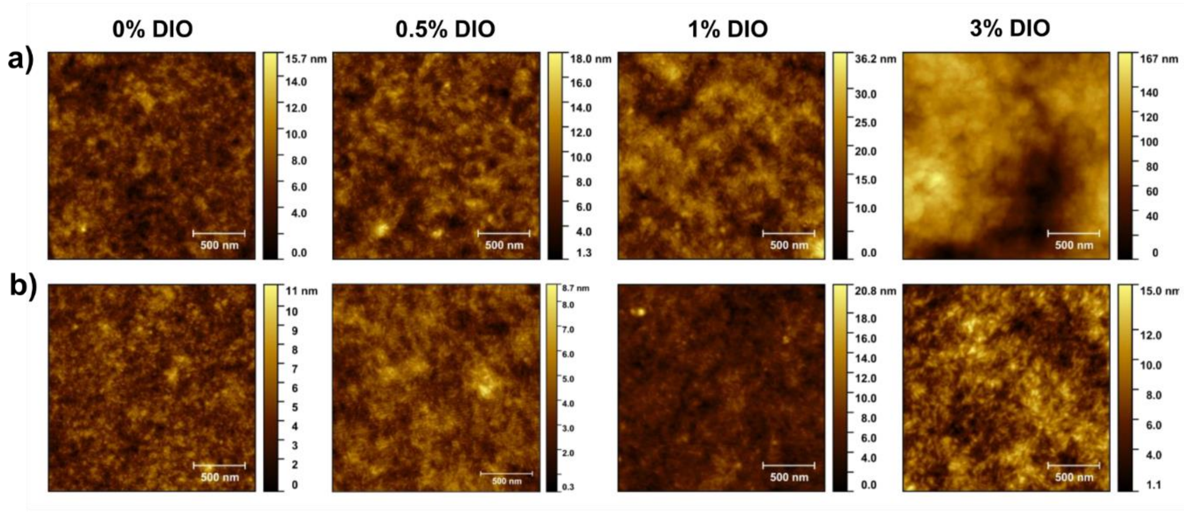

Figure S26: AFM height topography images of **(a)** PBDB-T:ITIC and **(b)** PBDB-T:$PC_{71}BM$ blend films processed with 0, 0.5, 1 and 3 Vol% DIO. The scale bar is 500nm.

Table S5. Root mean square (RMS) roughness values extracted from the AFM data shown in **Figure S25**.

DIO Concentration [Vol%]	PBDB-T:ITIC RMS roughness [nm]	PBDB-T:$PC_{71}BM$ RMS roughness [nm]
0	1.6	1.1
0.5	1.8	0.9
1	4.4	1.5
3	30.9	2.0

References

(1) Nečas, D.; Klapetek, P. Gwyddion: An Open-Source Software for SPM Data Analysis. *Open Physics* **2012**, *10* (1), 181–188. DOI: 10.2478/s11534-011-0096-2.
(2) Jacobs, I. E.; Wang, F.; Valdez, Z. I. B.; Oviedo, A. N. A.; Bilsky, D. J.; Moulé, A. J. Photoinduced Degradation from Trace 1,8-Diiodooctane in Organic Photovoltaics. *J. Mater. Chem. C* **2018**, *6* (2), 219–225. DOI: 10.1039/C7TC04358A.
(3) Mai, J.; Xiao, Y.; Zhou, G.; Wang, J.; Zhu, J.; Zhao, N.; Zhan, X.; Lu, X. Hidden Structure Ordering Along Backbone of Fused-Ring Electron Acceptors Enhanced by Ternary Bulk Heterojunction. *Advanced Materials* **2018**, *30* (34), 1802888. DOI: 10.1002/adma.201802888.
(4) Ohno, T.; Yatsuya, S. Growth of Fullerene Nanoparticles Prepared by the Gas-Evaporation Technique. *Journal of Materials Science* **1998**, *33* (24), 5843–5847. DOI: 10.1023/A:1004422703786.
(5) Staniec, P. A.; Parnell, A. J.; Dunbar, A. D. F.; Yi, H.; Pearson, A. J.; Wang, T.; Hopkinson, P. E.; Kinane, C.; Dalgliesh, R. M.; Donald, A. M.; Ryan, A. J.; Iraqi, A.; Jones, R. A. L.; Lidzey, D. G. The Nanoscale Morphology of a PCDTBT:PCBM Photovoltaic Blend. *Advanced Energy Materials* **2011**, *1* (4), 499–504. DOI: 10.1002/aenm.201100144.
